# Towards energy independence at KENTECH: A comprehensive microgrid implementation roadmap

**DOI:** 10.1016/j.heliyon.2024.e39131

**Published:** 2024-10-29

**Authors:** Lismer Andres Caceres-Najarro, Joonsung Jung, Yonggeon Lee, Seorin Yoo, Muhammad Salman, Jip Kim, Gyusub Lee, Youngtae Noh

**Affiliations:** a Department of Computer Engineering, Chosun University, Gwangju, Republic of Korea; b Korea Institute of Energy Technology (KENTECH), Naju, Republic of Korea; c College of Electrical & Mechanical Engineering, National University of Sciences & Technology (NUST), Islamabad, Pakistan; d Department of Data Science, Hanyang University, Seoul, Republic of Korea; e Department of Electrical and Computer Engineering, Seoul National University, Seoul, Republic of Korea

**Keywords:** Korea Institute of Energy and Technology (KENTECH), Microgrid, Building energy management system (BEMS), Energy efficiency, Net present cost (NPC), Investment cost, Operational cost, Photovoltaic (PV), Energy storage system (ESS), Campus energy management system (CEMS)

## Abstract

This paper introduces a comprehensive microgrid roadmap for the Korea Institute of Energy Technology (KENTECH), an energy specialized institute in South Korea, aligning with the country's overarching objective of achieving carbon neutrality by the year 2050. The roadmap outlines the integration of diverse energy resources—primarily renewables—to enhance sustainability and energy efficiency on campus. The paper also describes key elements for achieving autonomous energy operations through advanced technologies such as energy management systems, network gateways for system interoperability, static transfer switches, intelligent electronic devices, and power condition systems. It also presents details on our microgrid management system and customized data collection platform that acquires data from internet of things devices, advanced metering infrastructure, and automated revenue measurement. Additionally, by using a stochastic two-stage optimization model, we evaluate 1,320 scenarios to identify the optimal energy mix, focusing on the integration of renewable sources. Our findings reveal that a 15 MW solar capacity, coupled with an advanced energy storage system (ESS), can significantly enhance energy self-sufficiency, achieving up to 100% self-sufficiency in specific scenarios. The study demonstrates that increasing the solar installation from 5 MW to 15 MW, while integrating ESS, reduces dependency on imported power by 35% and enhances operational efficiency. Moreover, the optimization model indicates a potential 15% reduction in operational costs when demand response strategies are effectively implemented, particularly during peak activities. Furthermore, the microgrid design is projected to reduce CO2 emissions by up to 60%, contributing to KENTECH's role as a leader in sustainable energy management. These results highlight the roadmap's capability to transform KENTECH into a model of energy independence, with significant implications for similar institutions globally.

## 1 Introduction

Recently, the global climate crisis has heightened the urgency for significant reductions in greenhouse gas emissions. In light of the changing climate, many nations have united under the Paris Climate Agreement [1]. They have collectively pledged to limit the rise in global average temperature to no more than 1.5 °C above pre-industrial levels, striving to minimize this increase as much as possible. The nations involved in the Paris Climate Agreement have collectively committed to pursuing global carbon neutrality by the year 2050 [2], [3]. South Korea, a key participant in the global sustainability initiative, is committed to transforming its economy into a sustainable and green model by 2050 [4]. The country is actively progressing with energy efficiency strategies and targeted policies to cut down greenhouse gas emissions. This includes a focus on enhancing energy savings within the building sector^1^ and encouraging the adoption of clean energy and green initiatives through promotions and incentives.

Following the Russo-Ukrainian war, South Korea's system marginal price quadrupled over a span of 17 months. This significant rise is due to the fact that nearly 95% of the energy consumed in South Korea is imported, making the country's energy prices highly vulnerable to external events. This situation highlighted two key lessons: energy security is crucial for the power system, and energy prices in South Korea are extremely sensitive to global disruptions. To address this, increasing energy self-sufficiency has become a priority. Additionally, electricity prices have been steadily rising over the past two years. In response, the Korean government is now promoting distributed energy supply and has enacted the “Special Act on the Promotion of Distributed Energy” [5]. As a result, microgrids have emerged as a critical solution for enhancing energy self-sufficiency.

In South Korea, buildings within the academic sector, including universities and university hospitals, are substantial energy consumers. An analysis by the Korean Association for Green Campus Initiative on Seoul's top 25 energy-intensive buildings in 2022 revealed that these academic institutions comprised 40% of the total energy consumption [6]. Seoul National University (SNU) has been on top of the list as the most energy-consuming university since 2007, using 50,775 tons of oil equivalent (TOE) [7]. Korea University and Yonsei University are also among the highest energy consumers, ranked 15th and 16th, using 18,294 TOE and 18,514 TOE, respectively. The significant energy consumption in university buildings is attributable to the varied architectural designs of academic structures and the presence of laboratories and research facilities, which necessitate a constant and uninterrupted power supply.

The rapid expansion of smart buildings and electric vehicles (EVs) within modern energy systems has heightened the demand for sophisticated energy management strategies, particularly within microgrids [8], [9]. As these technologies become more integral to distribution systems, there is an increasing need for innovative approaches that optimize energy usage and enhance system flexibility. In response, recent research has developed multi-layered frameworks that effectively coordinate smart buildings, EVs, and microgrid scheduling. These frameworks leverage game-theoretic, bi-level, and robust optimization techniques to manage uncertainties, improve voltage stability, and reduce operational costs [10], [11], [12]. Moreover, decentralized coordination models have been proposed, incorporating energy communities and internet data centers into local congestion management strategies, thereby improving grid reliability and reducing reliance on traditional thermal units [13]. The integration of advanced demand response (DR) programs, coupled with dynamic tariff mechanisms, further enhances the resilience of local energy systems, particularly when these programs are aligned with renewable energy sources and energy storage systems (ESS) [14], [15], [16].

Additionally, the incorporation of carbon taxes and eco-environmental scheduling in multi-energy communities highlights the need for robust, flexible frameworks that can adapt to the evolving demands of modern energy markets [17]. To this end, nested and multilevel optimization frameworks can be employed to extract flexibility from distributed energy resources, thereby improving the coordination between transmission and distribution systems [18], [19]. The development of tri-layer stochastic frameworks for electricity market management, alongside two-stage optimization mechanisms for energy and ancillary services, emphasize the role of such optimization frameworks in supporting energy independence and enhancing system resilience [11], [20]. There are also various works on improving the efficiency of multi-energy microgrid operations. For instance, some enhance efficiency using dispatch and mixed-integer linear programming models [21], others assess low-carbon performance through convex reformulation [22], and some manage risk with a stochastic model that balances costs and other factors [23]. Collectively, these advancements illustrate the transformative potential of microgrids in achieving sustainability and energy independence, particularly within academic and research institutions that are increasingly adopting microgrid technology as they transition toward carbon-neutral campuses.

While these studies have contributed valuable insights into microgrid technologies, there remains a need for practical, comprehensive frameworks that can be adapted for institutional settings such as academic campuses. This paper seeks to address this gap by outlining a roadmap for microgrid development at the newly established Korea Institute of Energy and Technology (KENTECH), which aims to construct a microgrid utilizing diverse energy sources, with a primary focus on renewables for achieving enhanced energy efficiency within its campus. By utilizing a stochastic two-stage optimization model, the study evaluates 1,320 scenarios to identify the optimal energy mix, demonstrating substantial reductions in both carbon emissions and operational costs. Unlike many existing studies that focus on daily or yearly analyses, this study captures the full scope of long-term investment impacts and cumulative operational costs over extended periods. The paper also presents the integration of a data collection platform that incorporates internet of things (IoT) devices, advanced metering infrastructure, and automated revenue measurement systems. Additionally, the study introduces the envisioned microgrid energy management system (MGMS) and multiple building energy management systems (BEMS) as essential components for autonomous energy operations. These efforts collectively contribute to sustainability, foster academic collaboration, and promote environmental management in response to evolving energy demands. The main contributions of this work can be summarized as follows:

• The paper provides a detailed microgrid roadmap that can serve as a model for institutions aiming to achieve energy independence by strategically combining renewable energy resources with ESS and fuel cells to optimize self-sufficiency and reduce dependence on external power sources.
• An exhaustive evaluation of more than 1,320 scenarios is conducted to determine the optimal energy mix, ensuring energy stability, environmental sustainability, and economic feasibility by accounting for variability in power demand, renewable generation, fuel cells, and energy storage.
• This study offers a comprehensive evaluation of investment value using a 15-year net present value approach, in contrast to most existing studies that focus primarily on daily or annual operating costs.
• The paper details the design and implementation of a customizable data collection platform that integrates IoT devices and advanced metering infrastructure to collect real-time data for energy management. Such a system is crucial for enhancing demand management and facilitating the integration of renewable energy sources.

The practical implications of this work are significant. The findings show that a carefully optimized microgrid system can reduce carbon emissions by up to 60%, making a substantial contribution to environmental sustainability. Furthermore, by improving energy efficiency and incorporating flexible demand response mechanisms, the microgrid can reduce operational costs by 15%, particularly during peak demand periods, highlighting its economic viability. This paper advances the field by introducing a comprehensive and scalable microgrid framework specifically tailored for academic institutions. Utilizing a stochastic two-stage optimization model, the study evaluates over 1,320 scenarios to account for long-term operational and investment impacts, demonstrating the feasibility of integrating renewable energy sources to achieve energy independence.

## 2 Background

**Figure 1:**
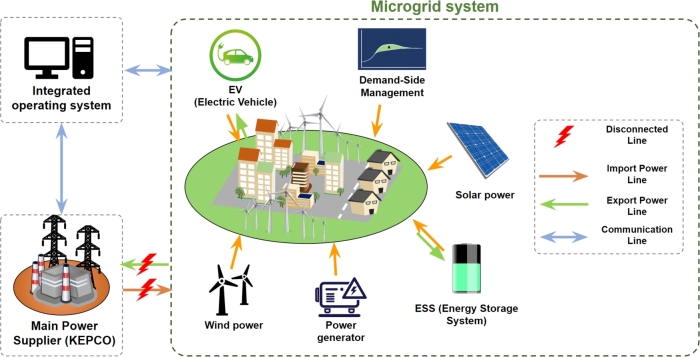
Conceptual diagram of a microgrid.

With the increasing deployment of distributed energy resources (DERs) and the rising frequency of extreme weather events, there is growing interest in microgrid technology, which enables more efficient and reliable grid operations. A microgrid is a localized power distribution network that manages the supply and demand of energy from DERs [24], see Fig. 1. Therefore, it can not only handle renewable energy resources but also effectively monitor and operate various energy sources existing within the grid, making its role even more critical in the future power networks. Microgrids can be broadly categorized into two main types: grid-connected and islanded.

**Grid-Connected microgrid:** Grid-connected microgrids are typically connected to the main power grid and interact with it during normal operation [25]. However, in case of emergencies or power disruptions, they can disconnect from the grid and operate independently. This type of microgrid is commonly used in applications such as buildings, campuses, and military bases. It aims to enhance the supply reliability for end-users and increase the efficiency of power supply. The grid-connected mode helps improve the stability and reliability of energy supply and allows for efficient utilization of local energy resources in combination with external energy sources, leading to cost savings [26].

**Islanded microgrid:** Islanded microgrids are designed to supply power to areas that are electrically isolated from the main grid, such as islands, deserts, remote regions, and polar areas. They are typically composed of a combination of resources, including diesel generators, wind turbines, solar power, and ESS [27]. The primary goal of islanded microgrids is to efficiently and continuously meet the local power demand using various energy resources and storage systems within the microgrid, without relying on external grid connections.

Table 1 summarizes the main characteristics of these types of microgrids. Nevertheless, it is worth mentioning that there are also cases where these two types can be switched. For example, it is possible to transition from a grid-connected mode to an islanded mode to ensure uninterrupted power supply during grid failures. This transition can be managed through the microgrid control system, taking into account environmental and economic factors.

**Table 1:** Comparison of grid-connected and independent microgrids.

Category	Grid-Connected Type	Islanded Type
Difference	Constant grid connection (operates independently in emergencies)	Always operates independently (in areas not connected to the grid)
Features	Uninterrupted power, electricity trading	Optimal combination of renewable energy
Target Areas	Buildings, campuses, military bases, factories	Polar regions, deserts, islands, remote areas, etc.
Purpose of Establishment	Improve supply reliability, pursue profit	Reduce supply costs, address environmental issues
Operational Constraints	Contracted power, power sales	Voltage/frequency maintenance, reserve capacity
Energy Sources	Gas, electricity, solar power	Diesel, solar power, wind power
Scale	Typically within 10MW	Varies depending on scale (several hundred to several thousand kW)

### 2.1 Microgrid component technologies

Microgrids are built to operate independently from the main power grid, using specialized technologies to manage power generation, distribution, quality, and emergencies [24], [28]. These systems optimize energy production, storage. As a result, they significantly improve the efficiency and reliability of the power system. They also enable seamless integration of energy sources and ensure a stable, resilient power supply.

**Power conditioning system** (**PCS**): PCS refers to power conversion systems that convert one or more of voltage, current, frequency and phase between the power source and the load [29]. In a microgrid, various energy sources exist, and PCS systems enable the integration of different energy sources, allowing for conversion between alternating current (AC) to direct current (DC) or DC/AC and adjustments of voltage, current, and frequency, depending on the intended use. Additionally, PCS systems control reactive power and improve power quality, making them suitable for use in both grid-connected and islanded microgrids.

**Figure 2:**
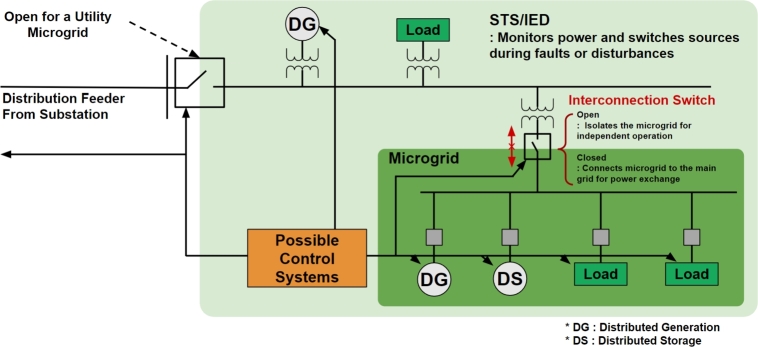
Microgrid power system configuration with the STS/IED.

**Static transfer switch**/**Intelligent electronic device** (**STS**/**IED**): STS/IED is a device that measures power at the interconnection point of the microgrid power system and, in the event of anomalies, securely disconnects the connected loads from the grid in order to provide stable power [30], [31]. As illustrated in Fig. 2, the STS/IED operates as a microgrid component that monitors for overcurrent and overvoltage protection. Should incidents occur or power quality deteriorates, the STS/IED engages the switch to safeguard the distribution system and guarantee continuous power provision.

**Figure 3:**
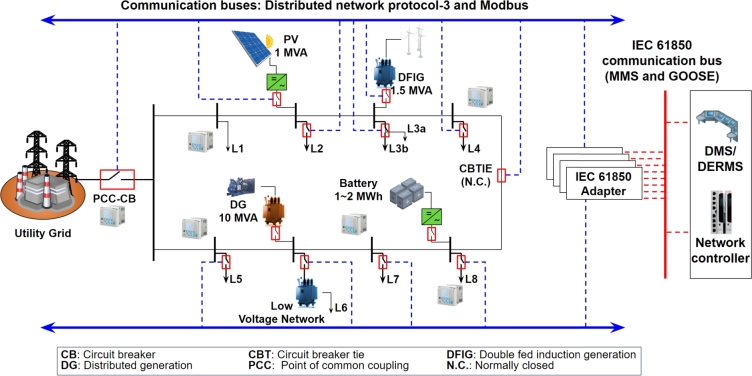
Example of communication protocols used in microgrids.

**Network gateway**: The network gateway in a microgrid is essential for managing and facilitating communication between the microgrid's internal systems and external networks. Leveraging the IEC 61850 standard, renowned for ensuring interoperability among diverse energy system components, it plays a pivotal role in translating and converting different communication protocols [32]. This functionality is vital for efficient control and integrated management of the microgrid, enabling crucial remote operations such as controlling energy production and usage, and resolving issues. The gateway's role in maintaining operational efficiency and reliability is indispensable, especially in adapting to varying power demands and conditions within modern energy systems. An example of such communication is depicted in Fig. 3.

**Energy management system** (**EMS**): The EMS is an advanced system of software and hardware designed to optimize and control power generation, storage, consumption, and distribution [33]. It plays a key role in managing power system operations, aiming to maximize energy efficiency and enhance microgrid stability. The EMS handles critical tasks such as energy forecasting, DER management, energy storage regulation, grid integration, data collection, and real-time monitoring. Depending on its application, the EMS may be referred to as home (HEMS), building (BEMS), or factory EMS (FEMS), each adapted to specific environments.

In addition to core technologies previously outlined, microgrids need supplementary elements for stable operation, such as seamless system separation [34], [35], resilience for disaster recovery [36], and tools for managing renewable energy integration [37]. Integrating advanced technologies like IoT, AI, data analysis, and energy quality management [38], [39], [40], [41] further enhances microgrid performance. This holistic approach is crucial for addressing the complex demands of modern microgrids.

### 2.2 Enhancing efficiency through microgrid

Microgrids offer several benefits, including reduced carbon emissions through renewable energy, lower energy costs, and a reliable, uninterrupted power supply. Academic campuses have complex load patterns due to their mix of educational, commercial, and residential buildings. Thus, campus microgrids should use smart algorithms for accurate energy forecasting and include a robust ESS to handle renewable energy variability. Reliable campus microgrids enable active participation in power transactions with the grid, turning campuses from power consumers into prosumers who can trade surplus power.

The concept of microgrids has recently gained prominence across various sectors, including university campuses, military bases, and hospitals [42]. The academic sector is well-suited for microgrid implementation, as it offers not just building space but also ample ground area to accommodate a variety of technologies. This makes microgrids a fitting solution for universities, meeting their need for a continuous power supply for critical educational and research facilities.

### 2.3 International trends in campus microgrids

The microgrid market was valued at $14.37 billion in 2021. Additionally, it is forecasted to grow at a compound annual growth rate 17.9% from 2022 to 2028, reaching $43.93 billion by 2028 [43]. Key players in microgrid technology, such as the United States (US), Australia, Japan, and China, are leading the industry.

Campus microgrids, in particular, are deployed most actively in the top universities in the US. They are primarily designed with the goal of enhancing power system resilience, particularly to mitigate the uncertainties of renewable energy-based distributed resources. The New York University (NYU) has developed an independent campus microgrid capable of isolating itself from the regional grid, ensuring stable operation even during extreme weather conditions [44]. Other educational institutions such as the University of California (UC) and the Illinois Institute of Technology (IIT) exploit microgrids to generate more than 90% of their total energy demand allowing them to operate independently most of the time [45]. These examples illustrate the significant role of microgrids in advancing energy-independence, decarbonization, enhancing energy stability, and ensuring reliability. This reflects a global trend towards sustainable and resilient energy solutions. Aligned with the global trend towards decarbonization and high energy utilization reliability, KENTECH, as a university aspiring to lead in the field of energy engineering, recognizes the importance of including microgrids as a fundamental requirement for advancing towards energy self-reliance.

## 3 Existing microgrids at universities

Microgrids at universities serve various purposes, from reducing the carbon footprint and energy costs to serving as living laboratories for students and researchers. They provide a unique opportunity to study energy management, renewable integration, and technological advancements in a controlled yet versatile environment. This section provides an overview of the current state and development of microgrids at universities around the world, offering a comprehensive perspective on how microgrids are adopted within academic environments.

### 3.1 Microgrids at US universities

Microgrids have been widely integrated into the U.S. energy infrastructure [46], [47]. Here, we focus on microgrids implemented within academic institutions. One of the oldest independent on-campus power generation systems was developed by NYU in the 1960s which was further enhanced in the 1980s with the installation of a large-scale Combined Heat and Power (CHP) plant [48]. As the CHP plant aged, it transitioned from oil combustion technology to natural gas generation and became a microgrid. The NYU microgrid is connected to the Con Edison distribution grid, purchasing electricity when demand exceeds its generation capacity. The microgrid of the NYU can also operate independently, isolated from the grid. It has been successfully tested during hurricanes, providing reliable power to the NYU campus during such events [44]. What sets NYU's microgrid apart is its unique location within the urban center of New York. This presented challenges related to limited land area for constructing various distributed power generation components, leading to a complex design process. The NYU operates a substantial 13.4 MW capacity CHP facility, which has the capability to supply both electricity and heat to 22 out of 37 buildings. Moreover, NYU microgrid employs two 5.5 MW natural gas turbines and a 2.4 MW steam turbine to generate power for the university, providing a diverse set of distributed power generation sources to meet the NYU energy needs [49], [50].

The Illinois Institute of Technology (IIT) operates AC-type microgrids that serve the campus demands. It integrates various distributed generation sources, including gas turbines, photovoltaic (PV) systems, wind turbines, and diesel generators [51]. The IIT has a peak power demand of 10 MW, and the installed capacity of distributed power generation facilities on campus microgrid is 9 MW, allowing the campus to operate independently from the main power grid for most of the time [52]. This led to cost savings of approximately $7,000,000 by reducing the need for substation upgrades and new substation installations. Furthermore, the IIT generates annual revenue of approximately $500,000 to $1,500,000 by providing ancillary services to the external grid [53]. The IIT manages the microgrid using distribution switches, smart meters, phasor measurement units, and BEMS. The microgrid in IIT is mainly composed of an 8 MW natural gas turbine power generation facility with two 4 MW Rolls-Royce gas turbine generators. It has an 8 kW wind power generation capacity and holds a total of 10 ESS with a capacity of 250 kW / 500 kWh, equipped with 50 kWh battery packs. Additionally, the ITT microgrid generates 140 kW solely from solar power through facilities on the roofs of three university buildings and a 20 kW solar installation on the roof of the canopy EV parking facility [52], [53].

The UC is largely self-sufficient, generating 92% of its electrical energy and meeting 95% of its heating and cooling needs internally. Its microgrid, with a peak load capacity of 42 MW, integrates a 3.8 million-gallon thermal energy storage system, combined heat and power (CHP) generation, a 3 MW solar facility, and a 2.8 MW biogas fuel cell system, which supplies 8% of the campus's baseline power demand [54]. The UC leverages big data from its supercomputer center to manage power demand, weather, and generation costs efficiently. Another U.S., based university named the University of Texas at Austin (UTA) operates one of the oldest self-sufficient energy microgrids, generating 100% of its heating and cooling energy via natural gas turbines. UTA produces 137 MW of power using natural gas and steam turbines, with excess energy sold to the grid through partnerships with Austin Energy [55]. This approach allows UTA to maintain a net-zero energy balance and contribute surplus power to the external grid.

Harvard University implemented various renewable energy sources across its Cambridge and Allston campuses, supplying power to over 250 buildings. The campuses have a combined maximum power demand of about 40 MW. The Harvard University operates a 5.6 MW CHP facility and has installed approximately 3 MW of distributed solar power generation facilities, in addition to two 10 kWh wind turbines. These facilities collectively contribute to the university's power supply, emphasizing sustainability and renewable energy generation as part of their energy portfolio [56], [57].

Princeton University's robust electrical infrastructure, featuring a 15 MW CHP facility as its cornerstone, enables the generation and distribution of power across its campus, ensuring reliability and cost-effectiveness. Complemented by a 4.5 MW solar power generation capacity and ESS, the university forms a resilient microgrid. This system's efficacy was notably demonstrated during Hurricane Sandy in 2012, when despite severe impacts in New Jersey, Princeton University maintained its steam, chilled water systems, and electrical supply, showcasing the resilience and sustainability of its microgrid [58].

Santa Fe Community College (SFCC) operates a microgrid integrated with 12.5 kW of solar power generation and a 100 kW battery ESS (BESS). Although its capacity is relatively small, it serves as an educational microgrid and can provide power as needed. Due to the initial success of this microgrid, SFCC is in the process of transitioning the entire campus to operate as a permanent microgrid. To achieve this, they have plans to install 1.5 MW of solar panels, a 770 kWh BESS utilizing 1.5 MW of solar generation capacity, and a 1 MW natural gas generator. These systems will be integrated with the existing educational solar array to support microgrid operations across the campus. Additionally, SFCC is planning a greenhouse nanogrid that combines advanced aquaponics and hydroponic technologies to efficiently manage water, energy, and recycling of waste materials [59].

Santa Clara University (SCU) is advancing its energy efficiency and sustainability goals through the installation of a smart microgrid. Such microgrid integrates weather forecasts with energy management, encompassing generation, transmission, distribution, and consumption, to optimize energy savings and provide real-time data on carbon emissions. This microgrid, expected to reduce energy consumption by 50% and save about 20% in energy costs, also enhances SCU's resilience to large-scale power outages, potentially supplying power to neighboring homes and businesses. Complementing this, SCU's microgrid is composed of a diverse array of renewable energy sources, including a 1 MW solar power facility, a 50 kW rooftop solar installation, and a wind turbine, which are capable of generating 1,500 kWh annually. Furthermore, it possesses California's largest rooftop solar thermal installation, with 60 solar thermal systems reducing water heating costs by up to 70% and cutting 34 tons of CO2 emissions annually [60], [61].

Oakland University is also a US based university that has a hybrid microgrid system that utilizes geothermal, solar, and combined heat and power technologies. This microgrid provides a sustainable and cost-effective energy solution for the university, that helps in reducing its reliance on the main power grid [62].

In addition to the universities previously mentioned, there is a growing trend of institutions implementing their own microgrids, including Eastern Mennonite University, Fairfield University, and Wesleyan University, among others. The adoption of microgrids is not only increasing in the US but is also gaining momentum globally. The following sections will provide details on universities in China, Australia, Korea, Japan, and Cyprus that have embraced microgrid technology. This reflects a broader shift towards sustainable and self-sufficient energy management in academic environments worldwide.

### 3.2 Micro-grids at Chinese universities

The Xiamen University (XU) has established a DC microgrid, incorporating a 150 kW capacity solar power generation facility and an energy storage facility, to enable direct and cost-effective management of renewable energy sources and building electrical loads via a web-based application. Through direct experimentation, the XU has determined that the efficiency of DC grids surpasses that of AC grids by 6∼8%, a finding attributed to reduced efficiency losses in areas like line losses and various load efficiencies [63].

Hefei University of Technology (HUT) has developed a microgrid that integrates a 70 kW solar facility, a 60 kW wind power facility, and a 5 kW fuel cell. This microgrid is notable for its two-layer control system, which includes local and central controllers. This design supports advanced research on system design, control, and scheduling strategies [64]. Similarly, another university in China named, Hangzhou Dianzi University (HDU) established a photovoltaic microgrid in 2007 in collaboration with Shimizu Corporation and under the auspices of Japan's New Energy and Industry Technology Development Organization and China's National Development and Reform Commission. This microgrid features a 120 kW PV facility with 728 solar panels, a 120 kW diesel generator, a 50 kW fuel cell, and a 150 kW BESS. Managed by a sophisticated power control system, the HDU microgrid maintains consistent load, voltage, and frequency even when operating independently from the main grid, demonstrating effective power quality control [65].

### 3.3 Microgrids at Australian universities

The microgrid of Deakin University (DU) at its Waurn Ponds campus features is designed with insights from those at UC and Princeton University, that not only provides independent power supply but also functions as a research hub for modeling, testing, and optimization. The microgrid at DU, currently meeting about 54% of the campus' power needs, incorporates a 7 MW ground-mounted solar power plant and a 2 MWh lithium-ion BESS. Additionally, it includes 23,000 solar panel units and is supplemented by an additional 250 kW of rooftop solar power generation across existing buildings, comprising 833 solar panels, along with a smaller 30 kWh BESS. Optical fibers are strategically laid underground from the solar plant to one of its buildings, and the solar plant's power lines connect to the western distribution line at high voltage, demonstrating an integrated approach to renewable energy utilization and infrastructure development [66].

Griffith University's Logan campus is undergoing a transformation towards a microgrid system with the installation of approximately 1 MW of solar panels across 5 buildings, currently fulfilling 73% of the campus's power demand. Future plans include the addition of another 4 MW of solar panels, aiming for a total of 5 MW in solar power generation facilities. A notable feature of this development is the “Sir Samuel Griffith Building,” renowned as Australia's first education and research facility to be completely powered by solar generation and hydrogen storage technology, incorporating both solar power facilities and a BESS for hydrogen energy storage [67].

Monash University (MU), in collaboration with the Australian Renewable Energy Agency, has developed a microgrid at its Clayton campus aiming to supply the majority of the campus's energy demand with renewable sources. The microgrid at MU has a total capacity of 3.5 MW and is supported by a 1 MW solar power generation facility and a 1 MWh ESS. Additionally, the campus features a 22 kW capacity EV charging station, enhancing its renewable energy infrastructure and facilitating EV charging and discharging [68], [69].

### 3.4 Microgrids at universities in other countries

The SNU, pioneering the first campus microgrid in South Korea under a national initiative led by the Korea Institute of Energy Technology Evaluation and Planning, has established three types of microgrid cells tailored to building characteristics, along with a cloud-based platform. This microgrid, particularly in key facilities like the bio-research center, is designed for up to four hours of independent operation during external power disruptions caused by natural disasters. The university's microgrid, which includes normal microgrids EMS elements in experimental buildings, features a 30 kW solar power generation and a 1 MWh capacity ESS. This system not only enables peak power and energy usage cost reductions, confirmed through comparative ESS analysis, but is also expected to save up to 20% on the university's electricity bills [70].

Chubu University in Japan, in partnership with Shimizu Corporation, has implemented BEMS across its campus. This microgrid integrates solar power generation, fuel cells, and cogeneration facilities, collectively supplying a total of 20 kW through a single power conditioning system. The cogeneration facilities operate continuously at rated output to minimize base load, contributing to the initiative's success in achieving a 15% reduction in power consumption [71].

In Italy, the University of Genoa at its Savona campus operates an advanced smart polygeneration microgrid that optimizes energy efficiency and management, primarily focusing on renewable sources. The microgrid is equipped with a system to monitor electrical, thermal, and mechanical facilities related to microgrids, checking the operational status of all elements and providing information in the event of issues or malfunctions. It includes a 115 kW PV solar power field, three concentrated solar power facilities with a combined capacity of 3 kWe/9 kWt, and three micro-turbines providing 160 kW of electricity and 289 kW of heat for co-generation. Additionally, an ESS supports its ‘E-car Operation Center’ platform, which facilitates grid-to-vehicle and vehicle-to-grid (V2G) operations. The ESS comprises a 1000 kWh NaNiCl2 battery and a 25 kWh lithium-ion battery, further enhancing the campus's commitment to sustainable and efficient energy utilization [72].

The university campus microgrid in Selangor, Malaysia, uses renewable energy sources like wind, biogas, solar, and hydrogen, with a battery for storage. This sustainable system provides reliable power, capable of generating up to 10,536,897 kilowatt-hours (kWh) of electricity per year [73]. Similarly, the Nanyang Technological University (NTU) microgrid in Singapore serves as a model for sustainable campus energy management by utilizing solar power and battery storage to optimize energy consumption and minimize reliance on the grid. It generates up to 6,000,000 kWh of electricity annually, which is equivalent to the average consumption of approximately 1,086 typical residential homes in Singapore [74].

In Canada, the Algonquin College conducted the energy service company (ESCO)^2^ project. Through the ESCO project, about 61% of the electricity that could be obtained from existing hydropower generation was saved. As a result, even if the electricity supply from hydropower generation was not possible, it became possible to supply electricity to the grid within the campus. The campus microgrid operates a total of 4 MW capacity from two 2 MW natural gas cogeneration units. As renewable energy sources, they have 500 kW solar power generation installed in two buildings at Woodroffe Campus and a 50 kW BESS installed at the Woodroffe Campus factory [75].

The University of Cyprus (UCY) operates a microgrid serving as a research and development platform for testing and experimentation in grid-connected and standalone modes. This microgrid infrastructure includes 30 kVA solar power generation facilities connected to the grid via inverters, a 10 kWh capacity BESS, smart meters, weather monitoring stations, and a HEMS. This setup enables the UCY microgrid to effectively test various energy management and renewable energy integration scenarios [76].

The University of Coimbra in Portugal has developed an experimental microgrid with an advanced energy system that integrates PV plants, lithium-ion batteries, EVs, and intelligent controllers. This microgrid is generating 1,466 MWh annually which meets around 22% of the campus's annual electricity demand [77].

## 4 Roadmap of KENTECH micro-grid

This section aims to construct an optimal energy mix that ensures energy stability, environmental sustainability, and economic feasibility. In order to account for various operational conditions and future uncertainties, we generated 1,320 scenarios for the generation planning model. Economic assessments were based on a 15-year recovery period and a 5% discount rate. The energy strategy includes a combination of central grid power, PVs, lithium-ion ESS, and fuel cells to maintain generation stability and meet peak demands. Optimizing the ESS charge/discharge schedules and energy source distribution maximizes efficiency in response to fluctuating power demands. This research provides a strategic framework for an energy mix that promotes the campus's long-term energy security and sustainability.

### 4.1 Scenario configuration

We consider 1,320 scenarios by carefully taking into account the following: 1) five different maximum installed capacity of solar power (2 MW, 5 MW, 10 MW, 15 MW, and 20 MW), 2) eleven self-sufficiency ratios (from 0% to 100%, increasing in 10% increments), 3) four seasonal variations (spring, summer, autumn, and winter), and 4) six daily patterns of power demand.

**Figure 4:**
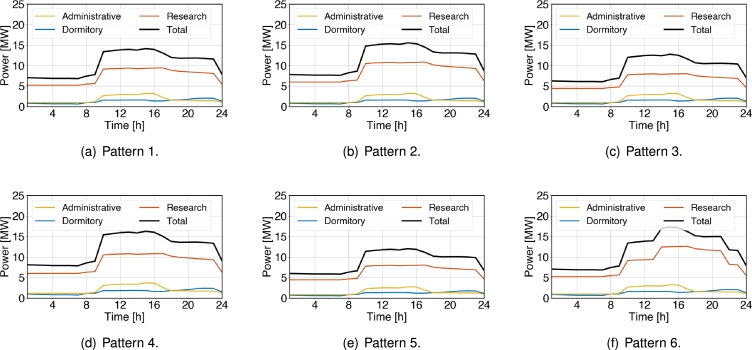
Considered six daily patterns of power demand: (a) Baseline, (b) increased demand in research building, (c) decreased demand in research building, (d) increased demand in overall campus, (e) decreased demand in overall campus, and (f) surge increase in research building at afternoon.

The six power demand patterns in a day are shown in Fig. 4. The lines in color black, blue, orange, and yellow indicate the total demand, dormitories demand, research buildings demand, and lecture buildings demand, respectively. We consider six different patterns by varying the demand in the dormitories, research buildings, and lecture buildings. Fig. 4(a) serves as a baseline for demand prediction, highlighting energy demand patterns for dormitories, research buildings, and lecture buildings. Building upon this, Fig. 4(b) presents a contrasting scenario with an increase in energy demand in the research buildings owing to the peak research activities. On the contrary, Fig. 4(c) illustrates the impact of energy-saving measures, depicting a decrease in demand within the research buildings. Fig. 4(d) presents a scenario of increased energy demand by 15% campus-wide, attributed to factors like enhancing comfort in typically unused rooms. Conversely, Fig. 4(e) shows the opposite scenario, considering a 15% reduction in overall campus energy demand. Finally, Fig. 4(f) highlights a notable surge in afternoon demand in the research building, attributed to specific experiments or events.

### 4.2 Power procurement resources used in the optimization model

The microgrid of KENTECH considers a variety of power procurement resources, combining different sources and methods to meet its future energy needs.

**Power from the main grid (Imported power):** In accordance with pre-existing electricity utilization contracts, the main grid's supplied electric power acts as the principal power source. It accounts for the aggregate electricity used by lecture halls, research buildings, and dormitories in adherence to zero energy building (ZEB) certification criteria [78]. Additionally, the main grid's power provision sets a benchmark for predicting and understanding fluctuations in energy demand.

**Solar power**: Solar power technology would be integrated to the KENTECH in two forms: Building integrated PV and building applied PV. Although solar power plays a vital role in improving power self-sufficiency, its installation capacity can be limited by the structural constraints of the building. Therefore, we consider a range of maximum installation capacities, from 2 MW to 20 MW, to accommodate different scenarios.

**Energy storage system**: To ensure flexibility and efficiency lithium-ion batteries for the ESS are considered. Additionally, to maintain the battery's lifespan and efficiency, its charging capacity would be restricted to a range of 20% to 80% [79]. The importance of the ESS is emphasized to enhance power self-sufficiency.

**Fuel Cells**: Fuel cells are characterized by their rapid responsiveness and stable power supply, ensuring a consistent and fixed output [80]. Therefore, KENTECH also considers fuel cells as part of its microgrid.

**DR**: DR strategies are key to improving both power stability and cost-effectiveness. The DR strategies primarily work by adjusting consumer power demand, rather than altering the actual power supply. This becomes especially significant in situations where power supply prediction is challenging.

**Electric Vehicles**: As EVs become increasingly common, KENTECH plans to install charging stations and examine the characteristics of their charging loads. The EVs have the ability to both charge and discharge power. Owing to their high power capacity, using them as a power source could affect the stability of the power network.

### 4.3 Optimization model for optimal power development

The two-stage stochastic optimization framework is employed for planning the generation mix [81]. In this framework, the problem is divided into two stages: planning and operation. In the planning stage, an investment strategy for the entire power development plan is formulated, with the goal of determining the investment decisions of generation resources while considering the long-term balance of power supply and demand. In the operation stage, an operation strategy for the actual power supply is developed based on the investment strategy determined in the planning stage. At this stage, scenarios such as varying load patterns and self-sufficiency ratios are considered to optimize the real-time operation strategy.

To co-optimize the operation and investment decisions simultaneously, the decision variables in the planning stage are modeled as recourse variables, allowing for the integration of the two stages in the optimization. This approach has the advantage of capturing the interaction between the investment and operational strategies in the development plan, leading to a more effective strategy. Formally, it is defined as

(1) $$\min_{\Xi }O^{C}≔[\gamma \cdot \underset{\text{Investment Cost}}{\underset{︸}{IC(p_{i}^{g,\max},e_{k}^{e,\max})}}+\sum_{s\in S}\omega_{s}\underset{\text{Operation Cost}}{\underset{︸}{OC_{s}(p_{ts}^{\mathrm{im}},p_{ts}^{\mathrm{ex}},p_{its}^{g}|p_{i}^{g,\max},p_{kts}^{\mathrm{ch}/\mathrm{dis}}|e_{i}^{e,\max},d_{nts},d_{ts}^{\mathrm{ev}})}}],$$

where *γ* denotes the capital recovery factor, while $\omega_{s}$ represents the probability of scenario *s*, acting as a weighting factor to calculate the expected operational cost $OC_{s}$. The decision variables are represented by $\Xi ≔[p_{i}^{g,\max},e_{k}^{e,\max},e_{kts},d_{nts},d_{ts}^{\mathrm{ev}}\geq 0,\;p_{ts}^{\mathrm{im}},p_{its}^{g},p_{kts}^{\mathrm{ch}},p_{kts}^{\mathrm{dis}}\in R]$. The investment cost, IC(⋯), and the operational cost for a specific scenario *s*, $OC_{s}(\cdots )$, are defined as

(2) $$IC(\cdots )≔C^{i,\mathrm{im}}p_{i}^{\mathrm{im},\max}+\sum_{i\in I}C^{i}p_{i}^{g,\max}+\sum_{k\in K}C^{e}e_{k}^{e,\max}$$

and

(3) $$OC_{s}(\cdots )≔\sum_{i\in I}C_{i}^{o}p_{its}^{g}+\sum_{n\in N}C_{ts}^{\mathrm{dr}}(D_{nts}-d_{nts})+C_{ts}^{\mathrm{im}}p_{ts}^{\mathrm{im}}-C^{\mathrm{ex}}p_{ts}^{\mathrm{ex}}-C^{\mathrm{ev}}d_{ts}^{\mathrm{ev}},$$

respectively. In (2) and (3), the parameters $C^{i,\mathrm{im}}$, $C^{i}$, $C^{e}$, $C_{i}^{o}$, $C_{ts}^{\mathrm{dr}}$, $C_{ts}^{\mathrm{im}}$, $C^{\mathrm{ex}}$, and $C^{\mathrm{ev}}$ represent the costs associated with each resource.

The minimization of the cost function $O^{C}$ in (1) involves the consideration of two primary costs, namely, the investment cost *IC* and operational cost for specific scenario $OC_{s}$. The *IC* consists of two key investment decision arguments: the first, $p_{i}^{g,\max}$, denotes the maximum generation capacity at the *i*-th node, and the second, $e_{k}^{e,\max}$, represents the maximum energy capacity at the *k*-th ESS. Furthermore, parameter *γ* is utilized to transform the *IC* into daily recovery costs, computed based on the total investment recovery period and the annual interest rate. On the other hand, the $OC_{s}$ is influenced by six arguments. The first two arguments are the imported power $p_{ts}^{\text{im}}$ and exported power $p_{ts}^{\mathrm{ex}}$ at time *t* in scenario *s*. The third argument represents the generated power $p_{its}^{g}$ at node *i* at time *t* for scenario *s*, conditioned by the maximum generation capacity. The fourth argument $p_{kts}^{\mathrm{ch}/\mathrm{dis}}$ denotes the charging / discharging power at the *k*-th ESS at time *t* in scenario *s*, conditioned by the maximum energy storage capacity. The fifth argument considers the demand $d_{nts}$ at building *n* at time *t* for scenario *s*. The last argument accounts for the EV demand $d_{ts}^{\mathrm{ev}}$ at time *t* in scenario *s*.

To solve (1) various constraints related to the supply-demand, DR, EV charging, and seamless operation are required. All these constraints are introduced to ensure the supply-demand balance in the face of uncertainty and to optimize the operation of the microgrid. In the following, details of such constraints are provided.

**Supply balance constraint within the microgrid:** The microgrid must continuously balance electricity supply and demand. The supply balance constraint

(4) $$\sum_{n\in N}d_{nts}=(p_{ts}^{\mathrm{im}}-p_{ts}^{\mathrm{ex}})+\sum_{i\in I}p_{its}^{g}+\sum_{k\in K}(p_{kts}^{\mathrm{dis}}-p_{kts}^{\mathrm{ch}}),\;\forall t\in T,\;s\in S,$$

formalizes the real-time supply-demand balance within the microgrid. This constraint ensures power generated, imported, and stored matches demand, minimizing losses and optimizing energy management The left-hand side in (4) represents the total demand for buildings (n∈N), while the right-hand side captures the total power supply, including net imported power from the grid ($p_{ts}^{\mathrm{im}}-p_{ts}^{\mathrm{ex}}$), internal generation $\left(\sum_{i\in I}p_{its}^{g}\right)$, and net energy storage discharge $\left(\sum_{k\in K}(p_{kts}^{\mathrm{dis}}-p_{kts}^{\mathrm{ch}})\right)$. This constraint applies across all time periods (t∈T) and scenarios (s∈S).

**ESS constraints:** The ESS constraints, defined as

(5) $$e_{kts}=e_{k,t-1,s}+(p_{kts}^{\mathrm{ch}}\cdot {ℵ}^{\mathrm{ch}}-p_{kts}^{\mathrm{dis}}/{ℵ}^{\mathrm{dis}}),\;\forall k\in K,t\in T,s\in S,$$

(6) $$0.2e_{k}^{e,\max}\leq e_{kts}\leq 0.8e_{k}^{e,\max},\;\forall k\in K,t\in T,s\in S,$$

(7) $$e_{k,t_{0},s}=e_{k,t_{24},s}=0.5e_{k}^{e,\max},\;\forall k\in K,t\in T,s\in S,$$

(8) $$0\leq p_{kts}^{\mathrm{ch}}\cdot {ℵ}^{\mathrm{ch}}\leq {\bar{P}}_{k},\;\forall k\in K,t\in T,s\in S,$$

(9) $$0\leq p_{kts}^{\mathrm{dis}}/{ℵ}^{\mathrm{dis}}\leq {\bar{P}}_{k},\;\forall k\in K,t\in T,s\in S,$$

regulate the state of charge (SoC), capacity limits, and charging/discharging rates to ensure efficient operation. In (5), we represent the relationship between the state of charge (SoC) of the energy storage at the current time interval and the previous time interval. Here, $p_{kts}^{\mathrm{ch}}$, $p_{kts}^{\mathrm{dis}}$ denote the charge and discharge power variables, and ${ℵ}^{\mathrm{ch}}$ and ${ℵ}^{\mathrm{dis}}$ represent the charging and discharging efficiencies, respectively. The minimum and maximum SoC limits for the energy storage system are defined in (6). To protect the battery, the system operates within the range of 20% ∼ 80% of its capacity. To ensure a balanced energy state, the initial and final SoC in each scenario are restricted to 50%, as specified in (7). Finally, (8) and (9) limit the charging and discharging power, ensuring they are less than the maximum output power ${\bar{P}}_{k}$, accounting for efficiency. These ESS constraints apply to all time intervals (t∈T) and scenarios (s∈S).

**DR constraint:** The DR constraint, defined as

(10) $$0.95D_{nts}\leq d_{nts}\leq D_{nts},\;\forall t\in T,s\in S,$$

is essential for managing peak loads and balancing supply and demand within a microgrid. For each of the research, administration, and dormitory buildings, it is assumed that the demand can be reduced by up to 5% from the estimated value $D_{nts}$. The compensation for demand response is modeled through the objective function's operational cost $OC_{s}$ as $C_{ts}^{dr}$. This constraint applies to all time intervals (t∈T) and scenarios (s∈S).

**EV charging constraints:** The EV charging constraints, defined as

(11) $$\sum_{t\in T}K^{\mathrm{ev}}D_{ts}^{\mathrm{ev}}=\sum_{t\in T}d_{ts}^{\mathrm{ev}},\;\forall t\in T,\;s\in S,$$

(12) $$0.85K^{\mathrm{ev}}D_{ts}^{\mathrm{ev}}\leq d_{ts}^{\mathrm{ev}}\leq 1.15K^{\mathrm{ev}}D_{ts}^{\mathrm{ev}},\;\forall t\in T,\;s\in S,$$

are intended to model the charging behavior of EVs. Equation (11) ensures that the total charging demand for EVs is maintained regardless of demand response over the entire time period. Here, $K^{\mathrm{ev}}$ represents the total number of EV chargers, and $D_{ts}^{\mathrm{ev}}$ denotes the estimated EV charging demand per time interval for each charger. The actual charging demand $d_{ts}^{\mathrm{ev}}$ is modeled to allow up to a 15% shift in response to demand as shown in (12), enabling flexibility such as charging load shifting. These constraints apply to all time intervals (t∈T) and scenarios (s∈S).

**Seamless operation constraint:** Seamless operation refers to the uninterrupted power supply provided by the generators within the campus microgrid. To enforce such operation, the seamless operation constraint, defined as

(13) $$\sum_{t\in T}[\sum_{i\in I}p_{its}^{g}+\sum_{k\in K}\left(p_{kts}^{\mathrm{dis}}-p_{kts}^{\mathrm{ch}}\right)]\geq K^{\mathrm{so}}\sum_{t\in T}\sum_{n\in N}\left(d_{nts}+d_{ts}^{\mathrm{ev}}\right),\;\forall s\in S,$$

is considered. In this study, the goal is to ensure that the proportion of energy supplied (left-hand side) from renewable sources such as solar, fuel cells, and energy storage systems is greater than the target seamless operation ratio $K^{\mathrm{SO}}$ for the total power demand on the right-hand side. The seamless operation ratio increases from 0 to 100% in 10% increments based on scenario compositions. This constraint applies to all time intervals (t∈T) and scenarios (s∈S).

We would also like to note that this study utilizes historical data on electricity demand from research, dormitory, and administrative facilities, along with rooftop solar power generation data, to generate forecast scenarios [82]. Additionally, the optimization problem presented in (1) is solved using GUROBI v10.0 on a personal computer with an Apple M2 Pro processor and 32 GB of memory. Our approach considers a variety of power procurement resources and methods to address future energy demands.

### 4.4 Power and energy mix according to energy self-sufficiency ratio

In this section, the results of simulations based on the proposed optimization model (1) are presented. We want to find the optimal investment decision and the corresponding configuration of energy mix infrastructure. We have conducted optimization simulations for a total of 1320 scenarios configured as described in section 4.1. Among them, only the results of four representative scenarios described in Table 2 are presented here due to space limitations.

**Table 2:** Details of representative scenarios.

Scenarios	Demand Forecast	Maximum Solar Capacity	Self-Sufficiency Ratio / Season	Remarks
1	Pattern 1	5 MW	0%∼100% / four	Baseline
2	Pattern 1	15 MW	0%∼100% / four	Solar capacity increase
3	Pattern 2	5 MW	0%∼100% / four	Increase in research building load
4	Pattern 4	5 MW	0%∼100% / four	Increase in total load

#### 4.4.1 Optimal investment decision results

**Figure 5:**
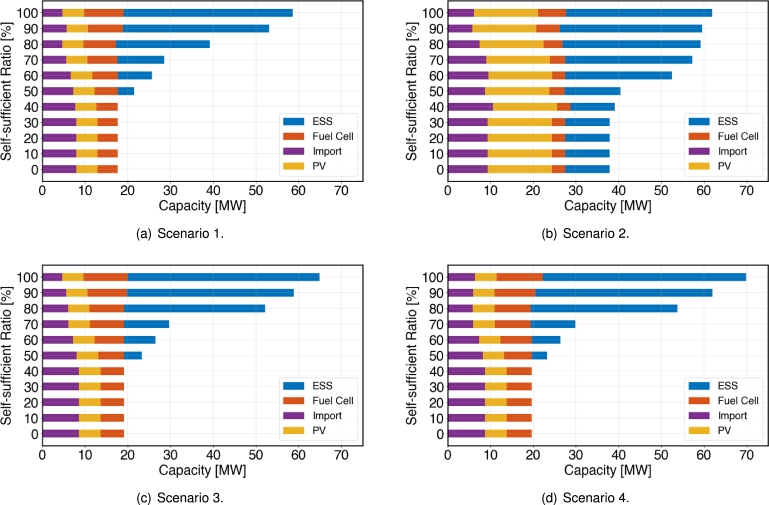
Power source composition according to the self-sufficiency ratio for the four representative scenarios.

Fig. 5(a) depicts the results for the scenario 1 based on the optimization model. It is observed that for all the range of the self-sufficiency ratio, the maximum allowable capacity of 5 MW for solar power is installed. This indicates that up to 5 MW of solar installation is economically viable under the given scenario conditions. Up to a self-sufficiency ratio of 30%, the same power mix is maintained, consisting of an imported power capacity of 7.92 MW and a fuel cell capacity of 4.77 MW. However, from a self-sufficiency ratio of 40% onwards, it can be seen that the imported power capacity is reduced, while the fuel cell capacity increases. Another interesting point is that energy storage installation does not occur up to a self-sufficiency ratio of 40%. However, from 50% onwards, there is a gradual increase in its capacity. This suggests that in situations with low self-sufficiency ratio, the flexibility within the campus microgrid, including changes in demand throughout the day, is supplied through imported power.

Fig. 5(b) depicts the results for scenario 2. Similar to the results obtained for the first scenario, it can be observed that solar installation is economically viable up to the maximum allowable capacity of 15MW, regardless of the self-sufficiency ratio. Additionally, up to a self-sufficiency ratio of 30%, the installation capacities of fuel cells and energy storage remain the same. However, from 50% onwards, there is a gradual increase in their capacities. However, unlike the results obtained for scenario 2, 10.38 MW of ESS installation occurs even when the self-sufficiency ratio constraint is set to 0% due to the increased of solar capacity. This indicates that a combination of solar and energy storage is more economically viable than increasing imported power to ensure a degree of self-sufficiency.

Next, Figs. 5(c) and 5(d) present the simulated results for scenarios 3 and 4, respectively. As before, it is observed that solar installation must be at its maximum capacity of 5MW to meet the self-sufficiency ratio constraint. In addition, when compared to scenario 1, it can be observed that in both scenarios 3 and 4, the installation capacities of resources have increased by 15% due to the rise in demand. Specifically, in scenario 3, only the research building load has increased by 15%, while in scenario 4, the entire load has increased by 15%. However, it's noteworthy that there isn't a significant difference in the installation capacities of energy resources.

Based on these results, it can be inferred that among the research buildings, administrative and lecture buildings, and dormitory, the research buildings are the most sensitive to changes in demand. This highlights the importance of DR resources that need to respond sensitively to changes in demand.

#### 4.4.2 Generation capacity based on the optimal investment

**Figure 6:**
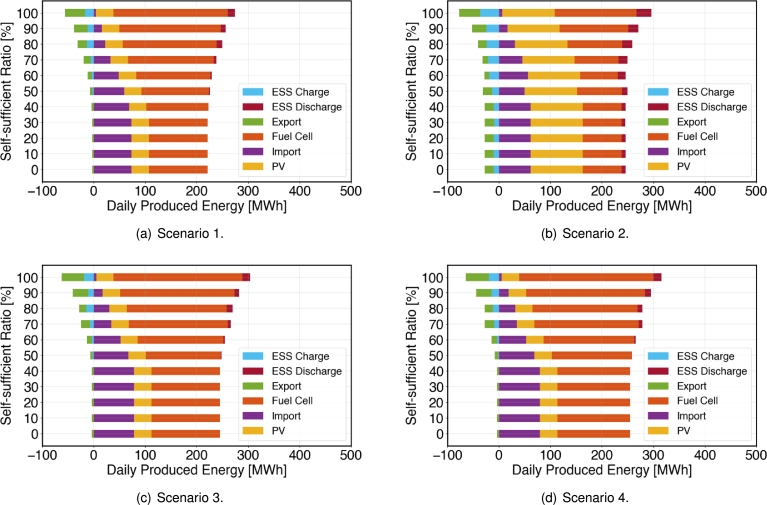
Generation capacity with respect to the self-sufficiency ratio for the four representative scenarios.

Fig. 6(a) shows the total generation capacity results for the scenario 1. It can be observed that for economically viable solar installations, the generated capacity remains constant regardless of the self-sufficiency ratio constraint. Conversely, for imported power, the quantity remains the same up to 30%, but gradually decreases from 40% onwards, with a significant increase in reverse power flow at 100% self-sufficiency ratio. Similarly, it's noticed that the generation capacity of fuel cells within the microgrid increases with installation capacity, along with an increase in charge and discharge capacity of ESS.

In Fig. 6(b), the daily generation capacity according to the self-sufficiency ratio constraint is depicted for the scenario 2. It is observed that, like in the case of scenario 1, the solar output capacity has increased, but it remains constant regardless of the self-sufficiency ratio. Up to 30% self-sufficiency ratio, the generation capacity of all power sources does not change generation capacity. Additionally, despite the reduction in the inflow (imported) power, the pattern of changes in the fuel cell output and inflow power remains consistent with variations in the self-sufficiency ratio. A significant aspect in this scenario is the operational involvement of ESSs which are present in the complete range of the self-sufficiency ratio. As the self-sufficiency ratio increases, there is a corresponding and gradual increase in the contribution of the ESSs. These results highlight the need for additional ESSs to enhance self-sufficiency.

Figs. 6(c) and 6(d) show the daily generation capacity with respect to the self-sufficiency ratio for the scenario 3 and 4, respectively. It is observed that the trends of daily generation capacity for both scenarios are similar despite the increase in power demand (load) for scenario 3 is only for the research building while total load is increased for scenario 4. With respect to the Scenario 1, the demand, which was previously 218.26, has increased to 241.62 and 251 for scenario 3 and 4, respectively. Interestingly, however, the solar PV generation remains constant regardless of the self-sufficiency ratio. Additionally, it is observed that up to a self-sufficiency ratio of 0 ∼ 30%, all power sources maintain the same energy mix. Due to the increase in demand, significant increases in resources other than solar PV are noted. However, when comparing Scenario 4, where the total load increased by 15%, with the Scenario 3, where only the research building's load increased by 15%, the increase is not substantial, with inflow power increasing by about 0.52 ∼ 1.3 MW and fuel cells by about 8.48 ∼ 10.46 MW. This indicates that the research building accounts for a significant portion of the total load and is sensitive to changes in self-sufficiency ratios.

### 4.5 Cost analysis by scenario

#### 4.5.1 Cost analysis method

For the cost analysis, we employ the NPC, which is widely recognized as a crucial metric in assessing the profitability of investments [83]. The NPC involves converting future cash flows into their present value by considering the time value of money. A discount rate is used in this conversion, and it is determined by reflecting various factors, such as the risk of a specific investment, the current market conditions, and other considerations. Typically, when an investment carries higher risks, the discount rate tends to increase, while it decreases in the opposite scenario. Specifically, a discount rate of 5% is applied based on a 15-year investment period for this study.

Table 3 provides a detailed breakdown of the costs of each power component calculated using the NPC method, with a self-sufficiency ratio of 70% and a maximum solar capacity of 10 MW. This table shows that fuel cells incur the highest daily operational costs, while solar and ESS prove to be relatively cost-effective during operation despite their high initial investment costs.

**Table 3:** Costs according to NPC (Self-sufficiency ratio: 70%, maximum solar installation capacity: 10 MW).

Category	Inflow Power	Solar	ESS	Fuel Cell
*IC*	$43,474.55	$9,287,500	$8,336,236.56	-
NPC per day	$11.48	$2,451.43	$2,200.35	-
Expectation of the *OC* per day	$8,484.64	-	-	$20,257.81
Total cost incurred per day	$8,496.11	$2,451.43	$2,200.35	$20,257.81

#### 4.5.2 Solar investment cost-benefit analysis

Table 4 summarizes the key parameters for the cost-benefit analysis of solar power generation. For this analysis, standard market price (SMP) and renewable energy certificate (REC) values were applied [84]. To account for the high volatility of REC, a synthetic weight of 1.02 was applied. This measure aims to enhance the accuracy of the analysis by considering the significant fluctuations in REC prices. As a result, the calculated electricity selling price was determined to be $ 0.1689. Normally, the solar modules performance tends to degrade over time. Thus, an solar annual module performance degradation rate of 0.7% was assumed. Other parameters such as the discount rate, system utilization rate, and average daily production hour are assumed to be 5%, 27.92%, 6.7 hours, respectively.

**Table 4:** Solar cost-benefit analysis settings.

Category	Value
SMP per kWh	$0.1125
REC per kWh	$0.05625
Electricity selling price per kWh	$0.1689225
PV Module degradation rate	0.7%
Discount rate	5%
System utilization rate	27.92%
Average daily production hours	6.7 h
Annual power production	24,455 MWh

**Figure 7:**
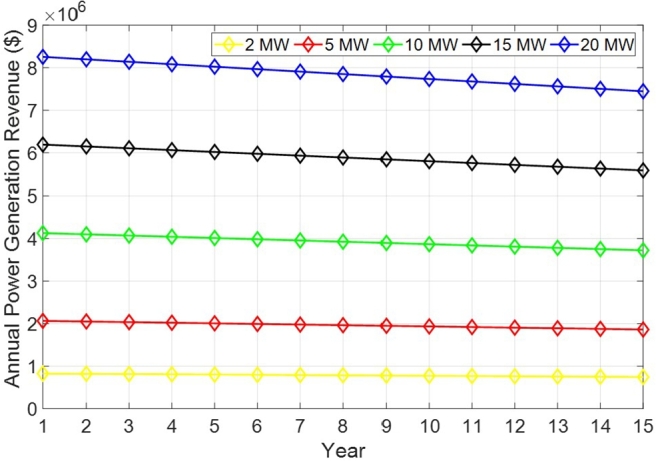
Annual power generation revenue with respect to the solar modules degradation rate for various solar power capacities.

Fig. 7 shows the annual power generation revenue for various solar plant capacities in a period of fifteen years under the assumptions considered in Table 4. In general, it is observed that as the generating capacity of a solar power plant increases, so does the revenue. Additionally, as the usage of the power plant increases the annual revenue decreases for all the generation capacities. However, the decrease in the annual revenue is more prominent for higher generation capacity. For instance, by comparing the first and last year, there is a lost in revenue of $81,129 and $808,900 when the generation capacity is 2 MW and 20 MW, respectively.

Table 5 provides a comprehensive cost-benefit analysis of solar power plants with various capacities, ranging from 2 MW to 20 MW. It is observed that the generation revenue increases significantly with capacity, indicating higher financial returns for larger installations. For instance, the revenue escalates from approximately $11 million for the 2 MW capacity to over $110 million for the 20 MW capacity. Conversely, the generation costs follow a similar upward trend, although the growth rate is proportional and consistent across different sizes, ensuring that larger plants do not disproportionately incur higher costs. The benefit, calculated as the difference between generation revenue and generation cost, also shows a positive correlation with the capacity of the solar plant. The generation cost, which represents operational or maintenance expenses, is considerably lower than the benefit, resulting in a favorable cost-benefit ratio. Remarkably, this ratio remains relatively stable at approximately 2.40 across the different capacities, highlighting the efficiency and economic viability of solar energy investment regardless of the scale.^3^

**Table 5:** Comparative cost-benefit analysis of solar plant capacities ranging from 2 MW to 20 MW.

Capacity	Generation Revenue	Generation Cost	Benefit	Cost	Cost-benefit Ratio
2 MW	$11,062,570	$3,926,580	$7,866,870	$3,266,020	2.41
5 MW	$27,601,275.00	$9,816,450.00	$19,627,958.25	$8,165,045.25	2.40
10 MW	$55,165,785.00	$19,632,899.25	$39,229,773.00	$16,330,089.75	2.40
15 MW	$82,748,678.25	$29,449,349.25	$58,844,660.25	$24,495,135.00	2.40
20 MW	$110,292,355.50	$39,265,799.25	$78,431,659.50	$32,660,179.50	2.40

#### 4.5.3 Investment feasibility for the installation of fuel cells, solar power, and ESS

Table 6 presents a thorough financial comparison among distinct energy generation sources. It is observed that traditional fuel sources, while yielding the highest generation revenue at $85,870,143.00, offer a relatively low cost-benefit ratio of 1.03, reflecting a marginal benefit over the costs incurred. In contrast, solar energy demonstrates a more favorable cost-benefit ratio of 2.40, attributed to its significantly lower generation costs and substantial benefits, thereby underscoring the efficiency and economic advantage of solar installations. The integration of ESS with solar resources slightly reduces the cost-benefit ratio to 1.65, suggesting that while energy storage introduces additional costs, it still maintains a considerable net benefit. Furthermore, the hybridization of solar and fuel with ESS yields a competitive generation revenue of $141,035,928.00, with an associated cost-benefit ratio of 1.21. This integration of resources reflects a synergistic benefit from diversifying energy sources, albeit at a higher operational cost.

**Table 6:** Comparative cost-benefit analysis of different energy generation sources.

Source	Generation Revenue	Generation Cost	Benefit	Cost	Cost-benefit Ratio
Fuel	$85,870,143.00	$73,384,153.50	$61,120,440.75	$59,137,869.00	1.03
Solar	$55,165,785.00	$19,632,899.25	$39,229,773.00	$16,330,089.75	2.40
Solar + ESS	$55,165,785.00	$27,135,512.25	$39,229,773.00	$23,832,702.75	1.65
Solar + ESS + Fuel	$141,035,928.00	$100,519,666.50	$100,350,213.75	$82,970,571.75	1.21

Although solar power yields the highest cost-benefit ratio in electricity production, it is important to note that this analysis assumed revenue generation based on average daily sunlight hours. In practice, however, actual power generation frequently underperforms relative to these theoretical averages due to variability in solar irradiance. To improve reliability and mitigate this discrepancy, the incorporation of ESS and fuel cells is an essential augmentation to the solar infrastructure even if the cost-benefit ratio is reduced.

#### 4.5.4 Analysis of carbon emission credits and renewable energy certificate revenue

Table 7 explores the revenue generated from the sale of carbon emission credits based on various solar power generation capacity. We have considered incremental prices from $7.5 to $30.68 per ton of CO2-equivalent (t CO2-eq) emissions credit. Additionally, the revenue from carbon emission credits created through solar power generation was calculated based on the carbon emission coefficient of 0.4434, designated by the Intergovernmental Panel on Climate Change in 2021 [85]. This table shows that the profit per day escalates with the solar capacity: a 2 MW installation yields a modest profit starting at $44.99 at the lowest carbon credit price and reaching $184.03 at the highest price point. This upward trend continues consistently, with the 20 MW installation securing a daily profit of $450.04 at the lowest price point, culminating in $1,840.66 at the highest price point. These results show the direct correlation between the scale of renewable energy deployment and the economic benefits derived from carbon credit sales. The larger the capacity, the greater the CO2 reduction and, consequently, the higher the potential revenue from carbon emission credits. This relationship highlights the dual environmental and economic incentives for investing in larger renewable energy projects.

**Table 7:** Daily profit from carbon emission credits at various price points.

Capacity	Generation	CO2 reduction	Daily carbon credit profit when carbon emission credit is				
	per day	[t CO2-eq]	$7.5	$9.75	$15.5	$22.5	$30.68
2 MW	13.53 MWh	5.99	$44.93	$58.40	$92.85	$134.78	$183.77
5 MW	33.83 MWh	15.00	$112.50	$146.25	$232.50	$337.50	$460.20
10 MW	67.67 MWh	30.00	$225.00	$292.50	$465.00	$675.00	$920.40
15 MW	101.50 MWh	45.00	$337.50	$438.75	$697.50	$1,012.50	$1,380.60
20 MW	135.33 MWh	60.00	$450.00	$585.00	$930.00	$1,350.00	$1,840.80

Table 8 presents the profit derived from the sale of reverse power at varying levels of self-sufficiency and SMP per kilowatt-hour (kWh). The SMP price was set at a base value of $0.19, and then we apply variation rates of ±15% and ±20% resulting in a range from $0.15 to $0.23 per kWh. In general, it is observed that there is a consistent profit across self-sufficiency ratios for each SMP increment, highlighting the potential financial stability offered by reverse power transactions. For self-sufficiency ratios of 0% to 30%, the profit remains unchanged, suggesting a threshold below which the revenue from reverse power sales does not increase. However, as the self-sufficiency ratio increases beyond this threshold, the profit shows a substantial increase. This is particularly evident at a 100% self-sufficiency ratio, where the maximum profits are recorded, highlighting the scalability of financial benefits with increased energy self-sufficiency.

**Table 8:** Reverse power sales profit at various self-sufficiency ratios and SMPs.

Self-sufficiency ratio	Reverse power	Profit with respect to standard market price per kWh				
(%)	(MWh)	$0.15	$0.16	$0.19	$0.22	$0.23
0	3.1	$465.00	$494.06	$581.25	$668.44	$697.50
10	3.1	$465.00	$494.06	$581.25	$668.44	$697.50
20	3.1	$465.00	$494.06	$581.25	$668.44	$697.50
30	3.1	$465.00	$494.06	$581.25	$668.44	$697.50
40	4.34	$651.00	$691.69	$813.75	$935.81	$976.50
50	5.15	$772.50	$820.78	$965.62	$1110.47	$1158.75
60	7.64	$1146.00	$1217.62	$1432.50	$1647.38	$1719.00
70	13.79	$2068.50	$2197.78	$2585.62	$2973.47	$3102.75
80	18.44	$2766.00	$2938.88	$3457.50	$3976.12	$4149.00
90	26.51	$3976.50	$4225.03	$4970.62	$5716.22	$5964.75
100	39.05	$5857.50	$6223.59	$7321.88	$8420.16	$8786.25

Table 9 shows the profitability derived from the sales of reverse power under varying self-sufficiency ratios, taking into account a combined pricing model that includes the SMP and REC weighted at 1.5 times. Similar to Table 8, price variations of ±15% and ±20% are considered. It is observed that in the range of 30% self-sufficiency and below, little variation in profits was observed. Additionally, the self-sufficiency ratio has a direct correlation with the profit realized from reverse power sales; higher self-sufficiency leads to increased profits. For instance, at a 0% self-sufficiency ratio, the profit commences at $674.25 for a combined price of $0.15 per kWh, whereas at a full 100% self-sufficiency ratio, the profit increases to $8,493.38 for the same combined price point.

**Table 9:** Reverse power sales profit at various self-sufficiency ratios considering fixed price contract, SMP, and REC.

Self-sufficiency ratio	Reverse power	Profit with respect to combined price (SMP+REC×1.5) per kWh				
(%)	(MWh)	$0.15	$0.16	$0.19	$0.22	$0.23
0	3.1	$674.25	$716.25	$1,085.63	$1,161.00	$1,011.38
10	3.1	$674.25	$716.25	$1,085.63	$1,161.00	$1,011.38
20	3.1	$674.25	$716.25	$1,085.63	$1,161.00	$1,011.38
30	3.1	$674.25	$716.25	$1,085.63	$1,161.00	$1,011.38
40	4.34	$943.95	$1,002.95	$1,179.94	$1,356.93	$1,415.93
50	5.15	$1,120.13	$1,190.13	$1,400.31	$1,610.50	$1,680.19
60	7.64	$1,661.70	$1,766.05	$2,076.37	$2,385.69	$2,496.00
70	13.79	$2,999.33	$3,186.79	$3,750.00	$4,313.21	$4,499.50
80	18.44	$4,010.70	$4,263.47	$5,013.38	$5,763.29	$6,016.04
90	26.51	$5,765.93	$6,126.30	$7,207.40	$8,288.50	$8,648.38
100	39.05	$8,493.38	$9,024.21	$10,616.72	$12,209.24	$12,739.06

#### 4.5.5 Cost analysis based on changes in demand

Here we focus our attention on the cost changes based on the patterns of energy demand fluctuations. The demand fluctuations patters considered here are those presented in Fig. 4 of Section 4. Table 10 shows the changes in the energy generation costs under the condition that the self-sufficiency ratio is maintained at 70% and the solar installation capacity is 10 MW. The results show that if the total demand increased by 15%, costs rose by 17.8%, while a 15% decrease in total demand led to a 17.6% reduction in costs. These results emphasize the importance of adjusting energy production and supply, and shows the need for an appropriate energy management strategy according to demand fluctuations.

**Table 10:** Cost analysis based on demand (Self-sufficiency: 70%, solar power capacity: 10MW).

Demand	Incurred costs per day					Cost	Ratio
fluctuation	Imported power	Solar Power	ESS	Fuel Cell	Total	per kWh	(%)
Pattern 1	$766.13	$220.65	$198.00	$1,823.18	$3,002.53	$137.75	100.00
Pattern 2	$843.18	$220.65	$205.35	$2,119.73	$3,387.53	$140.20	112.67
Pattern 3	$686.03	$220.65	$189.90	$1,530.60	$2,635.17	$120.38	87.38
Pattern 4	$870.78	$220.65	$205.28	$2,829.68	$4,472.13	$203.12	117.80
Pattern 5	$656.48	$220.65	$189.38	$1,408.42	$2,477.51	$113.41	82.31
Pattern 6	$914.25	$220.65	$253.95	$2,064.65	$3,447.67	$157.07	114.84

#### 4.5.6 Cost analysis based on changes in self-sufficiency ratio

Here, we analyze the cost implications of varying self-sufficiency ratios, specifically in a scenario where the maximum installation capacity of solar power is limited to 10 MW, and demand fluctuations are excluded. Table 11 presents the result of the cost analysis with respect to the variations of the self-sufficiency ratio. It is observed that not significant cost variance is noticed in the range of 0% to 40% of the self-sufficiency ratio. The initial increase in self-sufficiency primarily depends on the supply from the power grid, hence reducing the need for additional ESS. However, as the self-sufficiency ratio exceeds 50%, the required capacity of energy storage devices increases, leading to an associated cost rise. Achieving a considered level of self-sufficiency necessitates the expansion of ESS, resulting in increased total costs. These findings highlight the importance of balancing costs and self-sufficiency, as well as the need for optimal energy strategies.

**Table 11:** Cost analysis based on the self-sufficiency ratio (Demand variation: pattern 1, solar power capacity: 10 MW).

Self-sufficiency	Incurred costs per day					Cost	Ratio
Ratio (%)	Imported power	Solar Power	ESS	Fuel Cell	Total	per kWh	(%)
0	$10,239.75	$2,206.50	$138.75	$16,557.00	$29,141.25	$0.13	100
10	$10,239.75	$2,206.50	$138.75	$16,557.00	$29,141.25	$0.13	100
20	$10,239.75	$2,206.50	$138.75	$16,557.00	$29,141.25	$0.13	100
30	$10,239.75	$2,206.50	$138.75	$16,557.00	$29,141.25	$0.13	100
40	$10,239.75	$2,206.50	$138.75	$16,557.00	$29,141.25	$0.13	100
50	$9,716.25	$2,206.50	$723.75	$16,696.50	$29,343.00	$0.13	100.69
60	$8,892.75	$2,206.50	$1,356.00	$17,127.00	$29,582.25	$0.14	101.51
70	$5,330.25	$2,206.50	$2,304.75	$21,128.25	$30,969.00	$0.14	106.27
80	$5,330.25	$2,206.50	$2,304.75	$21,128.25	$30,969.00	$0.14	106.27
90	$2,610.00	$2,206.50	$2,422.50	$25,125.00	$32,325.00	$0.15	111.01
100	$874.50	$2,206.50	$2,529.75	$29,111.25	$34,722.00	$0.16	119.15

#### 4.5.7 Cost analysis based on changes in maximum solar installation capacity

Table 12 summarizes the cost analysis based on maximum solar power installation capacity for the scenario 1 taking into consideration a self-sufficiency ratio of 70%. It is observed that an increase in the installation capacity of solar power generation tends to increase the initial investment cost. This increase is due to the solar power installation cost rising proportionally with the capacity. However, there are advantages to this increased initial investment, most notably the reduction in electricity purchase costs and operational and maintenance expenses. On the other hand, an increase in generation capacity means an increase in the amount of power generated, which may necessitate the addition of an ESS. The ESS is required to ensure a stable power supply even when solar power generation is not possible. Therefore, determining the optimal ratio between solar power generation and ESS installation capacity is crucial to maximize economic benefits.

**Table 12:** Cost analysis based on maximum solar PV installation capacity (Scenario 1, self-sufficiency ratio: 70%).

Solar Power	Incurred costs per day					Cost	Ratio
Capacity	Imported Power	Solar Power	ESS	Fuel Cell	Total	per kWh	(%)
2 MW	$5,907.75	$441.00	$1,430.25	$28,590.75	$36,370.50	$0.17	100.0
5 MW	$6,161.25	$1,103.25	$1,051.50	$25,006.50	$33,323.25	$0.15	91.62
10 MW	$7,646.25	$2,206.50	$1,980.00	$18,231.75	$30,065.25	$0.14	82.66
15 MW	$8,587.50	$3,309.75	$2,825.25	$12,824.25	$27,546.00	$0.13	75.74
20 MW	$7,998.75	$4,412.25	$3,259.50	$9,774.00	$25,444.50	$0.12	69.96

## 5 KENTECH campus microgrid management system

The microgrid management system (MGMS) is an essential element in achieving efficient and cost-effective energy distribution within the microgrid. This system is a sophisticated and comprehensive software platform specifically engineered to manage the operation, control, monitoring, and optimization of the microgrid. Having a specific MGMS is deemed crucial in the context of campus microgrids. Campus microgrids can utilize a range of operational resources, including ESS, DR, and renewable energy sources. The MGMS plays a vital role in efficiently overseeing the energy, load, and grid connections inside campus microgrids. It facilitates the creation of optimal operational strategies to ensure efficient functioning.

**Figure 8:**
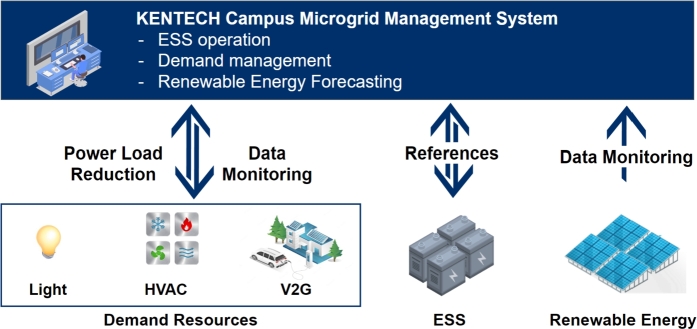
Overview of KENTECH campus MGMS.

Fig. 8 illustrates the functionality and concept of the MGMS that is being implemented at the KENTECH's campus. By establishing MGMS, KENTECH can analyze the power demand patterns of loads and demand resources like V2G to develop and manage optimal operational strategies. KENTECH's MGMS should ensure a balanced adjustment between energy demand and production within the campus, maximizing the efficiency of power usage. In that sense, several ESS and renewable energy sources will be key operational resources. The MGMS will operate these resources to achieve environmentally friendly and sustainable energy management through solar power generation, adjusting power supply as needed to enhance energy efficiency and reliability. The KENTECH's MGMS rather than be single large system, it is going to have a hierarchical structure that gradually increases from smaller units. The smallest unit is the BEMS, responsible for operating individual buildings.

### 5.1 Integrating BEMS into KENTECH

To set up the MGMS environment at KENTECH's campus, there are ongoing plans to implement the BEMS of the Korea Electric Power Corporation's (KEPCO) known as K-BEMS across all buildings on the campus. The K-BEMS capabilities would be further enhanced by incorporating controlling features according to the campus requirement [86]. The K-BEMS will collect data from various energy devices connected to both traditional power grid and new renewable energy sources. This initiative will involve upgrading building systems with high-efficiency equipment to replace older models. The main goal of these upgrades is to boost energy management efficiency and achieve cost reductions. In summary, K-BEMS will autonomously monitor and manage these systems, predict future energy demands, strive to reduce energy costs, and support sustainable and environmentally friendly energy management.

**Figure 9:**
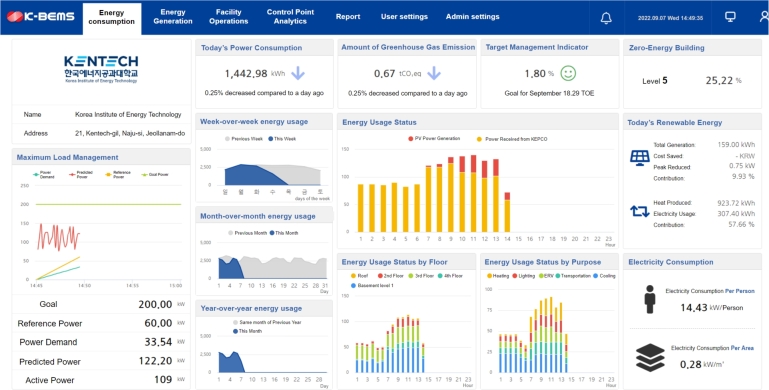
Example of MGMS implementation through K-BEMS.

The K-BEMS is currently installed on the third floor of the core facilities building at KENTECH. Fig. 9 provides an illustration of how KENTECH's energy management is monitored through K-BEMS. It monitors the operating conditions and outputs of all energy sources installed in the core facilities building. The K-BEMS at KENTECH provides insights into energy usage patterns of electrical loads, such as heating, lighting, and HVAC, within the building. This makes it easier for operators to manage and control these systems, giving them a clear view of how energy is used in the building.

### 5.2 Integrating CEMS into KENTECH

**Figure 10:**
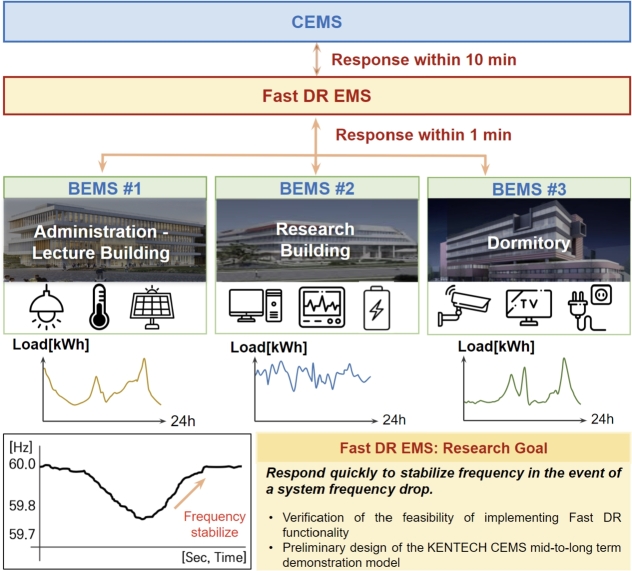
Conceptual diagram of CEMS.

In April 2023, KENTECH initiated research to establish community energy management system (CEMS) for three buildings on the campus as part of a national project funded by the KETEP. The CEMS (flexible energy CEMS, FeC-EMS) aims to develop and commercialize a sustainable energy community model capable of improving energy self-consumption rates, operational efficiency, and self-reliance within the power network of residential and commercial buildings. As shown in Fig. 10, the research concentrates on examining the patterns of energy usage in three distinct areas: the administrative and lecture building (BEMS #1), the research building (BEMS #2), and the dormitory building (BEMS #3). The main objective is to quickly identify and address any issues that arise, ultimately leading to the development of a more efficient and stable microgrid system. This rapid response is crucial for maintaining optimal energy management and ensuring the reliability of the power network.

#### 5.2.1 The CEMS at KENTECH

The research in sector-coupling operational technologies, including Plus DR,^4^ mainly concentrates on how power grids are operated by power exchanges. As a response to this, there is a growing need for technological investments and developments in various sectors, including technology design for community-level energy efficiency, operational standards, and inter-connectivity among diverse technologies. The microgrid at KENTECH incorporates the CEMS, which is an innovative energy operation system that is designed to enhance energy efficiency. The CEMS at the KENTECH's microgrid will employ a multi-energy sharing grid topology that incorporates a variety of distributed resources including sector-coupling facilities like power-to-heat (P2H), power-to-gas (P2G), and power-to-mobility (P2M), as well as idle energy supply facilities and demand resources. Such system is specially designed to optimize the use of a diverse energy sources and facilities, aiming to make campus energy management more efficient and sustainable by balancing supply and demand methods.

**Figure 11:**
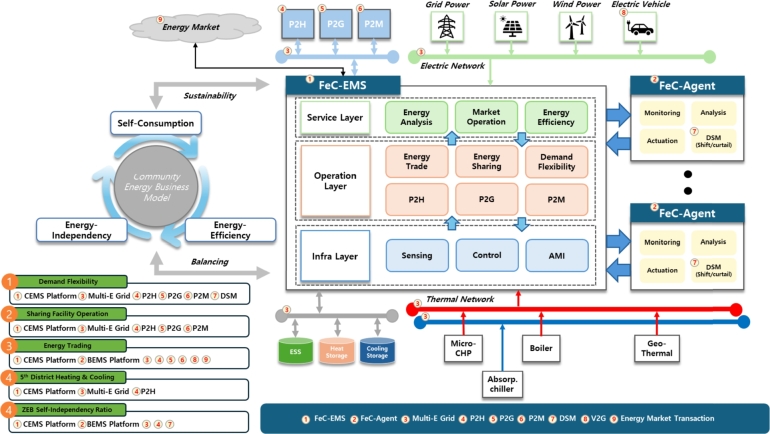
Detailed illustration of FeC-EMS.

Fig. 11 depicts the structural diagram of the CEMS that will be implemented at KENTECH. For achieving a balance and sustainable energy independence, energy efficiency, and self consumption, the CEMS at KENTECH incorporates nine elements: 1) a FeC-EMS comprising the infra, operation, and service layers, 2) BEMS platform with FeC agents comprising four processes, monitoring, analysis, actuation, and demand-side management (DSM), 3) multi electric network (Multi-E) grid which considers the electric, energy storage, and thermal networks, 4) P2H, 5) P2G, 6) P2M, 7) DSM, 8) v2G, and 9) energy market transaction. These elements are part of the five key technologies that the CEMS at KENTECH will embrace:

**1) Demand flexibility technologies:** This involves a variety of technologies, including distributed resources, sector-coupling technologies (e.g., P2H, P2G, P2M), energy storage solutions, technologies for harnessing underused energy, and DSM methods. These technologies allow for adaptable reactions to changes in energy demand and supply, improving the overall efficiency of energy operations.

**2) Shared facility operation technologies:** These technologies manage energy production, storage, and conversion facilities in the community using multi-energy sharing grids [88]. This leads to better energy self-consumption, operational efficiency, and self-sufficiency.

**3) Energy trading technologies:** These technologies facilitate two-way energy trading among buildings within the community with different ownership of energy supply facilities. They also enable energy trading among flexible energy communities, contributing to energy cost reduction.

**4) Fifth generation district heating an cooling technology:** This technology involves advanced district heating and cooling systems that use decentralized heat pumps [89], low energy systems [90], and network balancing technology [91].

**5) Zero energy building self-dependency ratio:** This involves achieving ZEB energy self-sufficiency by sharing and trading energy during the operational phase and carrying out pilot operations for ZEB certification at the community level [92].

## 6 Campus open energy platform

This section presents the development and deployment of a versatile, open energy platform specifically tailored for campus environments. By integrating innovative technologies and methodologies such as automated revenue measurement (ARM) systems, IoT [93], and advanced metering infrastructure (AMI) [94], the platform characterizes a holistic model for efficient and secure data collection. The data collection includes a vast amount of information that pertains to energy generation, particularly within power producing facilities, as well as the administration of energy transmission and distribution resources, consumer energy consumption patterns, variations in energy market prices, and ARM systems. This section not only highlights the technical aspects and architectural framework of the platform but also presents its pivotal role in fostering a culture of sustainability, academic collaboration, and environmental stewardship within educational institutions.

### 6.1 Energy big data

The collected data in the platform will be obtained from several sources at KENTECH, which can be grouped into four main categories: power, gas and hydrogen, meteorological, and system control data. In the following details of these categories of data are presented.

**Power data:** Power data primarily consists of data related to the using and production of electricity. This includes several metrics such as power consumption, energy generation, voltage magnitude, current magnitude, phase angles, frequency, and harmonic component. The effective use of power data is key to improving demand management strategies, enhancing load forecasting accuracy, and inspiring innovative business models in the energy sectors. For instance, such data can be exploited to enable energy use optimization, leading to significant waste reduction and efficiency gains. Additionally, it can be used for load forecasting, which predicts future energy needs, and for DR management, adapting power usage in real time to supply conditions. Such dual capacity of optimization and forecasting is essential for integrating renewable energy sources and ensuring grid stability [95]. By leveraging power data, utility companies and energy managers can make informed decisions, resulting in energy systems that are more efficient, sustainable, and aligned with the changing dynamics of energy consumption and production.

**Gas and hydrogen data:** Gas and hydrogen data, which includes details on hydrogen production, storage, transportation, usage, and potential leakages, is crucial in developing strategies to minimize emissions and environmental impacts [96]. Its significance is particularly important in the maintenance and stable operation of hydrogen production facilities, where continuous real-time monitoring helps preemptively identify and mitigate potential large-scale incidents. This proactive management approach, informed by a comprehensive analysis of gas and hydrogen data, is essential for ensuring safety and sustainability in the field of hydrogen energy, a focus of recent academic research in energy management and renewable energy sources.

**Meteorological data:** The role of meteorological data is integral to the reliable operation of EMS in microgrids, particularly those heavily reliant on renewable energy sources. Key metrics such as solar irradiance provide essential insights for predicting solar energy generation, with the intensity of sunlight directly influencing solar panel output [97]. Similarly, cloudiness impacts solar efficiency by indicating the extent of cloud cover. Temperature and humidity also play a crucial role in determining the operational performance of renewable energy systems [98]. For instance, extreme temperatures can affect the efficiency of both solar panels and wind turbines, while varying humidity levels impact their durability and electrical components [99]. Additionally, the meteorological data indirectly inform the energy demands of the system. For instance, changes in solar irradiance and temperature can alter the requirements for heating or cooling systems, thereby affecting overall energy usage. Precipitation (e.g., rain and snow) is another factor in managing renewable energy sources [99]. Rain can clean solar panels, potentially increasing their efficiency, but heavy snowfall might cover and reduce their output. The comprehensive analysis of these diverse weather-related data is crucial for maintaining the balance and efficiency of the microgrid. By closely monitoring and adapting to these environmental factors, the microgrid can optimize the use of renewable energy sources, ensuring a more sustainable and efficient energy management system.

**System control data:** This comprises mainly data from renewable energy generation systems, ESSs, and power semiconductors like circuit breakers and short-circuiters. Unlike conventional baseline generation systems such as thermal and nuclear power, which can maintain consistent production, renewable energy sources like solar and wind power are inherently variable [98], [99]. Therefore, a sophisticated control system, backed by comprehensive data on production patterns and environmental conditions, is indispensable for optimizing their output. The ESS data, including crucial parameters like state of charge, state of health, voltage, current, and impedance, is important for maintaining stability and efficiency, especially when integrated with fluctuating renewable sources [100]. Additionally, data related to power semiconductors, including forward and reverse characteristics, switching speed, power loss, and voltage and current ratings, play a fundamental role in ensuring the safe and efficient operation of the system [101]. Collectively, this system control data forms the backbone of a responsive and resilient campus microgrid, playing an important role in enabling optimized performance and advancing sustainability efforts.

### 6.2 Design and development of campus open energy platform

**Figure 12:**
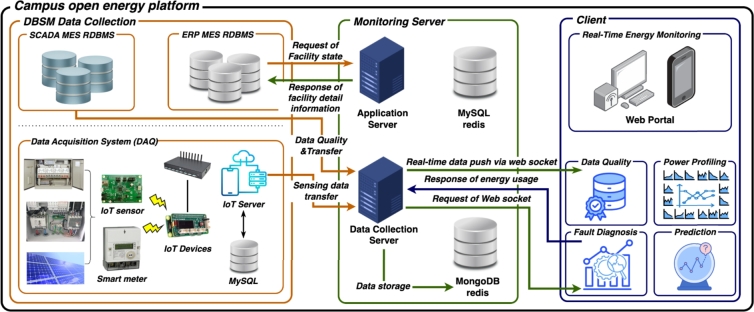
Data flow diagram for the campus open energy platform.

The data flow of the campus open energy platform comprises three interconnected layers: data collection based on database management system (DBMS), monitoring server, and client. As depicted in Fig. 12, the data collection based on DBMS utilizes smart meters and IoT sensor devices to gather power data and raw sensor data. The collected data is stored in a relational format in a relational DBMS (RDBMS), where tables are interconnected. While a DBMS stores and manages data in file format without inter-table relationships, an RDBMS stores and manages data in table format with inter-table relationships, allowing a more efficient data storage, organization, and management [102]. The data stored in the RDBMS is then transmitted to the monitoring server via MQ telemetry transport (MQTT) communication, where it supports various data processing functions such as data visualization, time-series analysis over specific periods, short-term and ultra-short-term demand forecasting, and fault diagnosis. Finally, clients can access useful data analysis and visualization results through a dashboard on the web via computer and/or mobile device.

#### 6.2.1 Architecture of the campus open energy platform

**Figure 13:**
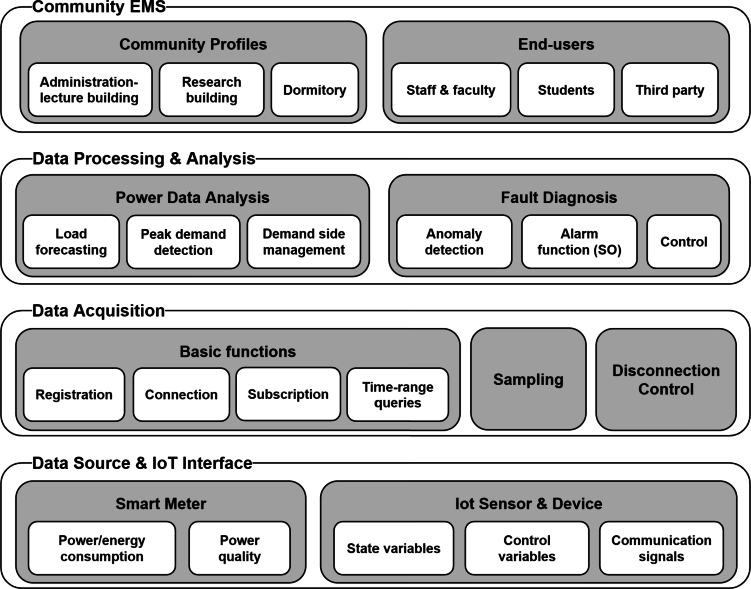
Structure diagram of the campus open energy platform.

As depicted in Fig. 13, the architecture of the campus open energy platform consists of four layers: 1) data source and IoT interface layer, 2) data acquisition, 3) data processing and analysis, and 4) dashboard CEMS. The data source and IoT interface layer involves collecting multi-modal data from various devices such as smart meters and IoT sensors and transmitting it to the cloud server. The data acquisition layer is defined as a network connection management module between smart meters, IoT devices, and the cloud server. The data processing and analysis layer includes functions for data processing used for load management (such as power demand forecasting, peak power analysis, and load flexibility analysis and identification) and fault diagnosis (anomaly detection, alarm processing, and control). Finally, the community EMS layer is defined by the power demand profiles of different campus community facilities (e.g., research buildings, administrative buildings, dormitories) and platform users (faculty, undergraduates, and graduate students, and third parties).

The campus open energy platform considers various modules within its architecture such as:

**Data collection module:** The data collection module collects raw data from the IoT sensor devices through the data management module, which is implemented in the sensor data collection server, see Fig. 12.

**Data storage module:** The data module employs the unified modeling language to efficiently manage a diverse range of data. This module defines the essential functions and variables for various classes such as the management portal, community, user, and data source, while also establishing the relationships among them.

**Data management module:** The data management module transmits and stores data collected from AMI, and provides a data retrieval function that enables the acquisition of data tailored to client requests.

**Data processing module:** It supports data processing applications for various system operations such as AMI data management, geographic information data integration, distribution system status estimation, load modeling improvement, solar systems, electric vehicles, time-varying load modeling, long-term planning, energy storage, visualization, DR, and real-time plan. This enables efficient and reliable campus microgrid operation.

**Dashboard module:** The dashboard module offers visualizations for data quality, analysis reports, demand patterns, and energy source comparisons. It provides real-time data quality monitoring, summarizes key power usage and weather data, and displays demand trends across different timescales. Additionally, it visually compares the proportions of various energy sources like solar, hydrogen, and ESS, enabling quick assessment of their usage in the community.

#### 6.2.2 Cloud deployment of the campus open energy platform

**Figure 14:**
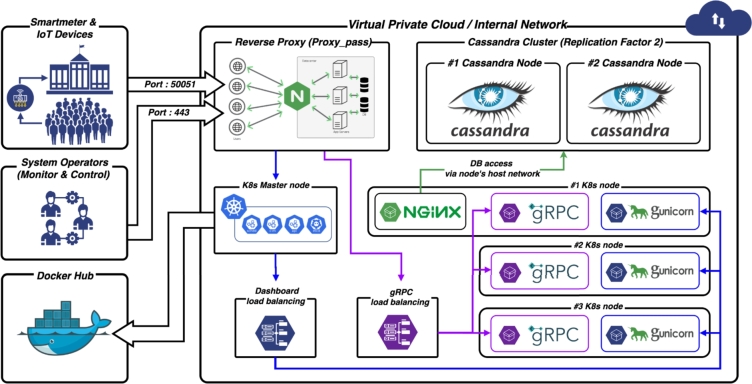
Campus open energy platform cloud deployment diagram.

As depicted in Fig. 14, the cloud deployment of platform leverages a NoSQL distributed database management system to construct a Cassandra-based database infrastructure [103]. This setup is complemented by the utilization of the Google remote procedure calls (gRPC) framework [104], ensuring server scalability and load balancing across multiple servers to mitigate individual server load. The platform is intended to be deployed via Naver Cloud [105]. Additionally, the platform architecture is designed around a two-node Cassandra-based columnar data store with a replication factor of two, data collection microservices based on gRPC launched on three Kubernetes nodes, and dashboard containers (i.e., gunicorn and django) initiated on three Kubernetes nodes. This structure achieves balanced data network traffic and dashboard functionality, facilitated by load balancing and ingress services. These services distribute data evenly across multiple servers, thus ensuring tangible scalability. Furthermore, to conceal the Kubernetes cluster, a reverse proxy is expected to be employed, launching it on a bastion server. This deployment strategy not only optimizes data flow and server efficiency but also aligns with the overarching goals of enhancing the platform's operational resilience and scalability.

## 7 KENTECH campus microgrid demonstration roadmap

**Figure 15:**
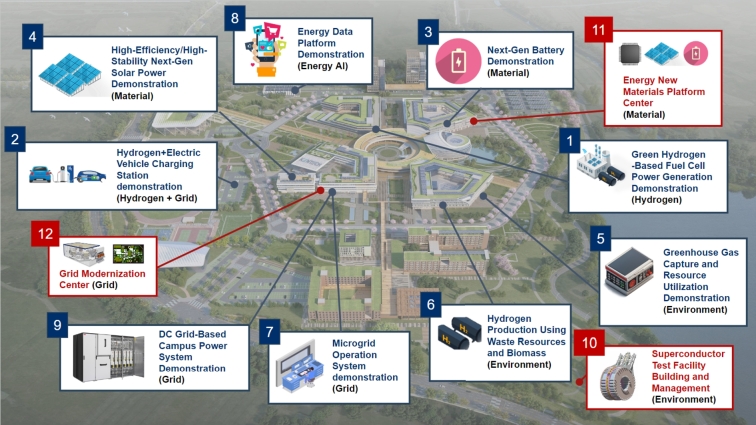
KENTECH campus microgrid demonstration areas.

This section presents the roadmap for the campus microgrid demonstration, detailing the plans and objectives within five priority research areas, namely, energy AI, energy advanced materials, next-generation grid, hydrogen energy, and environmental climate technology. These research areas are envisioned to transform the KENTECH campus into a specialized testbed. Furthermore, the demonstration in these five research areas can be broadly categorized into two parts: verification of component technology and energy facility verification for operation. These two categories comprise of 12 distinct components all together, as illustrated in Fig. 15.

### 7.1 Verification of component technology

The verification of component technology includes the establishment and management of a superconductor testing facility within the environmental climate technology domain. Furthermore, it also includes the establishment and administration of centers for energy material and next-generation grid.

**Figure 16:**
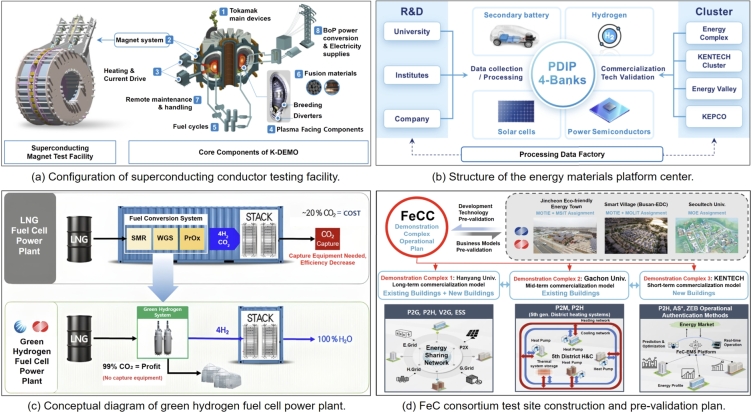
Energy facility verification for operations.

**Superconducting wire testing facility:** The superconducting wire testing facility at KENTECH is planned to be established over six years from 2022 to 2027, with a target budget of $33.6 million. This facility will allow to examine the quality of superconducting conductors and optimize their performance through the development of standardized testing and measurement procedures. Additionally, this facility is aimed at creating a center dedicated to verifying and producing superconducting magnets up to 16 Tesla, a key component in advancing artificial solar technologies. The facility is presently in the design phase. Fig. 16(a) shows the proposed layout for the testing equipment which is mainly composed of eight elements: 1) Tokamak major devices [106], 2) magnet systems, 3) heating and current injection devices, 4) plasma-facing materials, 5) fuel cycle devices, 6) fusion materials, 7) remote maintenance devices, and 8) balance of plant power conversion and electrical supply devices.

**Energy materials platform center:** The energy materials platform center is planned to be established over five years, from 2022 to 2026, with a budget allocation of $29.4 million. The plan includes setting up a comprehensive data processing center to support the full spectrum from research and development to commercialization. This center will assess the practicality, performance, economic feasibility, and market fit of technologies, aiming to enhance their applicability in real-world scenarios. The center will gather and process data obtained from research and development activities in the areas of secondary batteries, hydrogen, solar cells, and power semiconductors. To fulfill its goals, the center intends to develop the necessary equipment and establish a collaborative platform between industry and academia for the validation of new technologies. The energy materials platform center would evaluate the economic feasibility, reliability, and market demand of these technologies to assess their suitability for commercialization. The structure of the energy materials platform center is depicted in Fig. 16(b).

**Next-generation grid center:** The next-generation grid center is scheduled to be established over 5 years from 2023 to 2027, with a total budget of $20.8 million allocated for its development. The center aims to build design and testing facilities for wide bandgap semiconductor equipments, power semiconductors, and power conversion systems. These testing facilities are designed to foster the development of high-performance power electronics and circuits based on power semiconductors. The main goal is to create a testing center for full lifecycle technology development in next-generation grids. This includes validating technology for technical operations and stability through grid-connected tests, simulating power semiconductor equipment, and evaluating power conversion technology performance. The goal is to enhance the practicality and efficiency of existing and new technologies, focusing on sustainable societal and industrial growth through energy-efficient use.

### 7.2 Energy facility verification for operations

The energy facility verification for operations includes several key components, namely, green hydrogen-based fuel cell power generation, hydrogen and electric vehicle charging infrastructure, next-generation battery validation, next-generation solar panels, greenhouse gas capture technologies, and hydrogen production from waste resources and biomas. Moreover, it considers the development of specialized charging stations for hydrogen and electric vehicles, focusing on the hydrogen energy and next-generation grid sectors. In the following, details of these projects are presented.

**Green hydrogen-based fuel cell power generation:** With the urgent need for sustainable and green energy due to ongoing global warming, hydrogen is becoming a key source of future clean energy. However, conventional hydrogen production methods, such as gray hydrogen, rely on fossil fuels, which results in significant carbon emissions^5^ [107]. Therefore, as depicted in Fig. 16(c), our focus is on the production of green hydrogen, a sustainable alternative that does not result in carbon emissions, as opposed to the generation of conventional hydrogen. Green hydrogen is produced through the electrolysis of water using electricity generated from renewable energy sources such as solar and wind power [108]. Then the green hydrogen would be transformed into electricity through the use of fuel cells. Such a solution is not only sustainable but also environmentally friendly.

**Hydrogen and electric vehicle charging infrastructure:** We plan to systematically design smart charging infrastructure for hydrogen and EV charging within the microgrid. This includes the integration of hydrogen energy with the next-generation grid. The stations will be equipped with a hydrogen supply system, robust security measures, and diverse charging interfaces tailored for a range of scenarios. Furthermore, KENTECH will develop and technically validate real-time charging algorithms through simulations. In these algorithms, key considerations will include charging time, speed, and the battery's state of charge. Using these algorithms as a foundation, KENTECH will conduct tests on the actual charging infrastructure to confirm its efficiency and operational effectiveness. After validating and confirming the effectiveness of the smart charging stations through these tests a demand resource on campus that would significantly reduce energy costs will be established.

**Next-generation battery validation:** Energy material researchers are engaged in developing next-generation batteries, aiming for advancements in rapid charging, extended lifespan, and high energy density capabilities. KENTECH has established a dedicated specialization track in energy materials to accomplish these objectives. The development of specialized protocols to improve the capabilities of high-speed charge is currently underway. To prolong the life of batteries, algorithms for aging mitigation are being developed [109], [110]. These algorithms employ thermal and electrical control mechanisms and will be verified on real batteries. Researchers are currently dedicating their efforts to enhance the capacity of energy storage through the development of improved electrolyte formulations and electrode materials [111], [112]. To ensure the batteries' stability in challenging outdoor conditions, they will undergo comprehensive testing, which will place particular emphasis on environmental variables such as temperature, humidity, and vibrations. Enhancements would be made to the battery design and manufacturing processes in order to improve performance and safety.

**Next-generation solar panels:** The goal is to achieve improved energy conversion efficiency through high-efficiency and high-stability solar power generation systems, thereby increasing actual energy production. To enhance energy conversion efficiency, researchers in the field of energy materials are actively advancing solar panel technology [97], [113], [114]. This includes the development of perovskite solar cells and the improvement of PV efficiency through nanotechnology and innovative materials [115], as well as development of more efficient energy collection methods [116]. Furthermore, they are focusing on optimizing the design of solar power generation systems, taking into account various factors such as topography, solar irradiance, and incident angles [117]. To realize the goal of creating higher-stability solar panels, KENTECH's approach involves simulating their operation in outdoor environments to assess their performance. Based on these simulations, KENTECH will implement enhanced systems and accurately predict their performance in real-world operating conditions.

**Greenhouse gas capture technologies:** The study of greenhouse gas capture technologies is a rapidly evolving field, focusing on innovative methods like absorption, separation, transportation, and utilization of greenhouse gases [118]. This field includes prominent examples such as carbon capture and storage [119], direct air capture [120], and bioenergy with carbon capture and storage [121]. These technologies would be crucial in mitigating emissions from large-scale sources and directly from the atmosphere, representing a significant step forward in environmental conservation efforts. The objective is to implement greenhouse gas capture technologies effectively. In the future, through chemical reactions and electrochemical methods, KENTECH will focus on converting greenhouse gases into various forms of energy or chemical substances. Additionally, KENTECH will be implementing a system designed to manage and monitor each step of this process methodically. This forward-thinking approach will aim to ensure the efficiency and efficacy in reducing greenhouse gas emissions, contributing significantly to environmental sustainability.

**Hydrogen production:** KENTECH is planning to establish the hydrogen production process utilizing waste resources and biomass. Biogas is a byproduct generated through anaerobic digestion of organic waste resources such as food waste, sewage sludge, and livestock manure [122]. It primarily consists of methane (50-65%), carbon dioxide (25-50%), and small amounts of hydrogen, hydrogen sulfide, and other components [123], [124]. Currently, around 20% of surplus biogas produced in domestic anaerobic digestion facilities is not utilized and is either incinerated or released into the environment [124], [125]. To address this issue, KENTECH aims to develop hydrogen production processes that make use of waste gasification technology and biogas hydrogenation facilities [126]. The goal is to achieve both economic and environmental benefits through efficient hydrogen production from these waste resources and biomass.

### 7.3 Demonstration of microgrid system operation

This section outlines the demonstration specifics of CEMS at the KENTECH, which includes the design of the CEMS verification environment as well as the approaches used in CEMS load modeling and data acquisition.

#### 7.3.1 The CEMS at KENTECH

The development and demonstration of the CEMS for the KENTECH campus microgrid is currently underway, which is supported by the KETEP. The CEMS at KENTECH, a national project initiated in April 2023, is set to continue until December 2026, covering a duration of 3 years and 9 months. This project is led by the renowned Korean GS Engineering & Construction Co., Ltd.

The objective of the project is to create demonstration sites that will help in the early commercialization of the FeC-EMS. As illustrated in Fig. 16(d), this endeavor will be carried out in three distinct phases with a total budget of $12 million: short-term, medium-term, and long-term commercialization models. In the short-term commercialization model demand flexibility, P2H, and ancillary service technologies will be implemented. The medium-term commercialization model focuses on a fifth generation local heating and cooling system, which will be implemented in existing buildings using P2M and P2H technologies. In the long-term commercialization model, energy circulation systems will be implemented incorporating technologies such as P2G, P2H, V2G, ESSs, and biogas.

**Figure 17:**
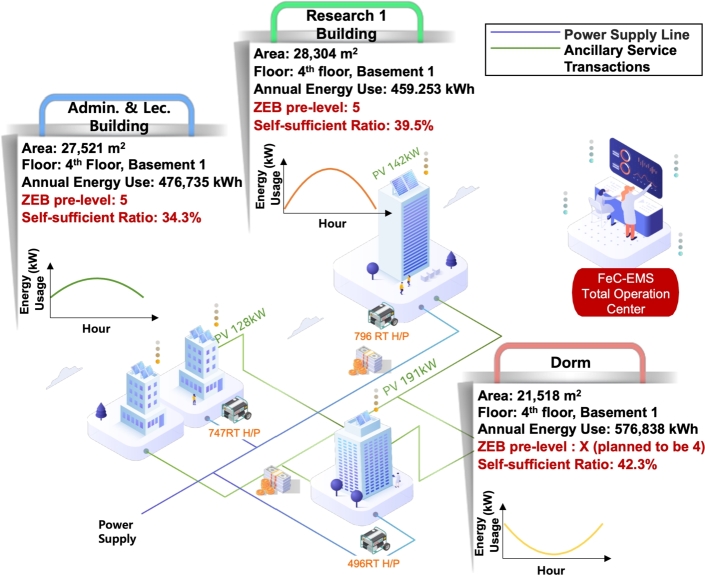
Internal power network configuration plan at KENTECH.

FeC-EMS is set to primarily provide frequency resources to the power grid, as illustrated in Fig. 17. The FeC-EMS at KENTECH aims to contribute to frequency control, which is achieved through load response. To accomplish this, four key verification facilities, including solar power generation, geothermal systems, BEMS, and FeC-EMS, will be utilized. These facilities are expected to increase energy self-sufficiency and enhance efficiency. For solar power generation, both rooftop solar panels and building integrated PVs are included in KENTECH's microgrid roadmap. Meanwhile, geothermal systems will be operated using efficient geothermal heat pumps, contributing to the project's overall effectiveness and sustainability.

#### 7.3.2 CEMS verification environment design

In the context of discovering grid-connected demand resources and conducting FeC verification, KENTECH is performing research related to investigating energy supply sources for each community, analyzing demand resources, and verifying auxiliary service provisioning functions. Additionally, the basic design of the FeC-EMS verification model for short-term FeC-EMS commercialization, verification of the possibility of providing FeC-EMS fast DR functions, case studies, and concept definitions, as well as the development of basic models for building demand patterns by usage type, are currently being carried out.

First, KENTECH is systematically investigating the energy supply sources for each community and conducting an analysis of demand resources. In this regard, research and analysis are ongoing for each verification target area, including legacy energy resources, energy supply system configurations (such as grid diagrams and piping layouts), building types, and flexibility resources.

Additionally, KENTECH is conducting the foundational design of a short-term operational FeC-EMS verification model for the effective operation of ancillary service provision functionalities. In this context, KENTECH is currently analyzing the energy status of the verification site, developing an additional resource portfolio, defining the concept and structure of the verification site, and designing the interconnection structure between the fast DR operational module and FeC-EMS of KENTECH.

Furthermore, KENTECH is conducting research to assess the feasibility of the fast DR functionality within the FeC-EMS. This involves conducting a preliminary study on estimating flexibility resources in renewable energy-based power grids, analyzing the characteristics and constraints of fast DR for providing flexibility, defining the functionality of CEMS fast DR, analyzing cases of maintaining reliability through fast DR in the event of power grid incidents, and conducting economic feasibility analysis of fast DR to secure flexibility in renewable energy-based power grids.

Lastly, KENTECH is conducting research on the development of basic demand patterns for buildings based on their usage. In this regard, KENTECH is conducting a preliminary study on models related to power demand patterns, obtaining and analyzing preliminary datasets for predicting demand by building usage types (i.e., research, administration, and residential), developing baseline demand models for buildings by usage type, and standardizing datasets for demand predictions by building usage type.

#### 7.3.3 CEMS load modeling and data acquisition

KENTECH plans to conduct research related to the discovery of grid-integrated demand resources and flexible energy community verification. This includes quantifying and analyzing demand resources for each community, detailed design of the new integrated FeC-EMS model, designing infrastructure for measuring demand in various building types, collecting real measurement data, and conducting research on inconvenience-based building load and resource potential models for the application of new integrated FeC-EMS algorithms.

First, there are plans to quantify and analyze demand resources for each community. In this regard, based on the demand resources identified in the first year, quantitative assessments of demand resources will be conducted according to building types in the target verification areas. An analysis of the actual impact of these demand resources will also be performed.

Additionally, there are plans to proceed with the detailed design of the new complex FeC-EMS verification model, alongside to design the infrastructure for building demand measurement by purpose and collecting actual measurement data. Efforts will be made to secure and establish the necessary infrastructure for both projects, which includes network architecture design, equipment procurement, and hardware system construction. This process will involve the campus demand measurement infrastructure architecture design and analysis of building demand patterns by purpose and load profiling within the campus. Additionally, the project will encompass an analysis and modeling of price elasticity of electricity demand based on building demand patterns.

Lastly, there are plans to conduct research on developing models for building loads and resource potential based on discomfort for the application of the new comprehensive FeC-EMS algorithm. In this regard, probabilistic modeling techniques will be developed through scenario analysis and characteristics analysis of demand prediction scenarios for individual buildings, and the potential capacity of fast DR resources based on historical data will be derived considering uncertainty. In addition, responsive models for fast DR considering uncertainty by demand type and strategic models for reduction discomfort by usage purpose such as work, residence, welfare, etc., are also planned for development.

### 7.4 Demonstration of energy data platform

The KENTECH campus electrical safety platform is an initial model of the KENTECH campus energy platform, planned for 2024 as part of the energy technology evaluation institute's project. It is currently under review for approval, and if passed, it is expected to be officially announced in the first half of 2024, with budget discussions ongoing. This platform is designed to acquire data related to electrical safety for energy facilities such as EV charging stations, ESS, and solar power generation facilities. Through this platform, remote electrical safety management will be conducted.

**Figure 18:**
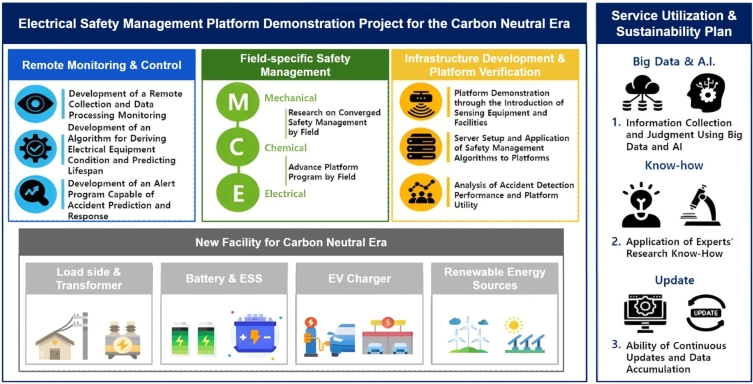
Campus demand measurement infrastructure architecture.

The conceptual diagram of the electrical safety platform validation project is shown in Fig. 18. The envisioned platform has three main roles: remote data monitoring, microgrid management, and accident prediction. Within the microgrid, various components such as batteries, ESS, electric vehicle chargers, and renewable power sources are included, requiring a platform capable of continuously processing various data. This role is fulfilled by the electrical safety management platform, which aims to create an environment for real-time monitoring of data. Additionally, the platform is designed to include algorithms for understanding the condition of electrical facilities, predicting equipment lifespans, and providing accident prediction and response capabilities, thus ensuring stable equipment maintenance. The platform, built with servers and safety management algorithms, will be subjected to analysis and verification of sensing device and equipment accident detection performance, as well as platform utility. In terms of the ongoing and future utilization of the platform, considerations include utilizing big data and AI for information collection and decision-making, incorporating research expertise, and maintaining continuous updates and data accumulation.

### 7.5 DC grid-based campus network demonstration

In the next-generation grid specialization area, there are plans to demonstrate a campus network based on a DC grid. This is one of the technologies related to smart grids and intelligent energy management systems, where a DC grid refers to a power grid system that uses DC to transmit and supply power [91]. Such a system can be deployed in the form of microgrids within buildings or industrial facilities and can efficiently manage the flow of power by connecting to distributed energy resources. The goal is to establish a campus network based on this DC grid through practical demonstrations within the campus.

## 8 Conclusion

This paper proposed a microgrid roadmap for KENTECH, drawing on the strengths of existing microgrid models. We identified critical components—such as power conversion systems, static transfer switches/intelligent electronic devices, network gateways, and energy management systems—to ensure efficient and autonomous operation of the campus microgrid. To provide a pathway toward an optimal microgrid configuration, we utilized a two-stage stochastic optimization model to assess resource investment and operation costs. Specifically, our analysis covered 1,320 scenarios with variations in solar power capacity, self-sufficiency ratios, seasonal changes, and demand patterns. The model optimized both the investment in energy resources, including photovoltaic panels, energy storage systems, and fuel cells, as well as their operational efficiency, with the objective of minimizing total costs and maximizing energy stability. The results indicate that increasing solar capacity from 5 MW to 15 MW reduces power imports by 35% and CO2 emissions by 60%. However, achieving self-sufficiency ratios higher than 50% necessitates additional energy storage system capacity to maintain stability. Furthermore, the cost-benefit analysis showed that solar installations have the highest economic efficiency with a ratio of 2.40, and integrating energy storage system provides a positive net benefit (1.65), highlighting the importance of a balanced approach to investment in renewable energy and storage resources for sustainable microgrid development.

This study also introduced a novel microgrid management system, built around three key innovations. First, campus energy management system is designed to optimize energy consumption across three building types: administrative lecture, research, and dormitory. It is envisioned to use advanced technologies such as power to heat, power to gas, and power to mobility to enhance efficiency and sustainability. Second, the system is supported by a cloud-based open energy platform for real-time data collection from diverse sources (e.g., power, gas, meteorological, and system control) and makes this information publicly accessible to foster transparency, research, and innovation. Third, a comprehensive field study is planned to validate the system's performance, involving the demonstration of pioneering energy technologies across five key areas—energy AI, advanced materials, next-generation grids, hydrogen energy, and environmental climate technology—positioning KENTECH as a dynamic testbed for pioneering energy solutions.

Our future work will focus on expanding the energy mix of the KENTECH microgrid by incorporating additional clean energy technologies, such as wind power, biomass, and other renewable sources, to further diversify the energy portfolio and enhance overall system resilience. We will also investigate the long-term reliability and robustness of the microgrid under extreme weather conditions to refine operational strategies and ensure sustained energy independence. Additionally, we plan to integrate the proposed data collection platform with a real-time control system, improving decision-making accuracy and enabling adaptive operational strategies within the campus microgrid framework. These efforts will contribute to a more resilient, flexible, and sustainable energy system, supporting both national and global goals for carbon neutrality.

## CRediT authorship contribution statement

**Lismer Andres Caceres-Najarro:** Writing – original draft, Methodology, Formal analysis. **Joonsung Jung:** Data curation. **Yonggeon Lee:** Resources. **Seorin Yoo:** Formal analysis, Data curation. **Muhammad Salman:** Writing – review & editing, Methodology, Formal analysis. **Jip Kim:** Formal analysis. **Gyusub Lee:** Validation, Investigation. **Youngtae Noh:** Supervision, Methodology, Conceptualization.

## Declaration of Competing Interest

The authors declare that they have no conflicts of interest regarding the research work presented in the paper entitled “Towards Energy Independence at KENTECH: A Comprehensive Microgrid Implementation Roadmap,” submitted for publication in the Elsevier – Energy and Buildings. There are no financial, personal, or professional relationships that could be perceived as constituting a potential conflict of interest. This research is conducted with full transparency and adherence to the ethical standards of scholarly publishing.

## Footnotes

1 Implementation of cost-effective technologies in new buildings, as well as retrofitting older buildings with these same energy-efficient technologies.

2 https://www.naesco.org/esco/.

3 A cost-benefit ratio higher than 1 is considered economically viable.

4 Plus DR is a program aimed at minimizing the control over renewable energy output, thereby contributing to the overall stability of an energy system [87].

5 For example, producing 1 ton of gray hydrogen emits 10 tons of carbon dioxide.
